# Insights into the Ir-O redox dynamics of an operating PEM water electrolyzer using *in operando* Raman spectroscopy

**DOI:** 10.1038/s42004-026-02162-9

**Published:** 2026-08-06

**Authors:** Sebastian Speer, Sven Jovanovic, Christina Oeß, André Karl, Eva Jodat, Rüdiger-A. Eichel

**Affiliations:** 1 Institute of Energy Technologies—Fundamental Electrochemistry (IET-1), Forschungszentrum Jülich, Jülich, Germany; 2 Institute of Physical Chemistry, RWTH Aachen University, Aachen, Germany; 3 Faculty of Mechanical Engineering, RWTH Aachen University, Aachen, Germany

**Keywords:** Electrocatalysis, Raman spectroscopy, Hydrogen energy, Catalytic mechanisms

## Abstract

*In operando* Raman investigations of iridium-based oxygen evolution (OER) catalysts in water electrolysis offer insights into catalytically relevant species and pathways, enabling optimization towards effective use of iridium. This work builds upon existing academic studies by utilizing industrially relevant proton exchange membrane catalyst-coated membrane electrode assemblies in an applicable cell design. To overcome challenges in spectral analysis, a methodology for spectral deconvolution with a focus on stability and validity was developed and verified by two-dimensional correlation analysis. Based on *in operando* studies during potential-dependent electrolysis and catalyst relaxation at open circuit potential, the oxygen evolution reaction pathway on iridium-based catalyst-coated membrane electrode assemblies was found to involve a concerted redox reaction of both iridium and oxygen. Formation of the active species occurs from iridium oxo-hydroxides and crystalline IrO_2_ via oxidative charging. Enhanced proton conduction due to ionomer presence in application-oriented systems promotes deprotonation, causing oxidative charging of the catalyst well before the oxygen evolution reaction onset.

## Abstract (web summary)

In operando Raman studies of iridium-based oxygen evolution reaction (OER) catalysts offer insights into relevant species and pathways, enabling process optimization for the effective use of rare metal resources. In this work, the authors use industrially relevant proton-exchange-membrane catalyst-coated-membrane electrode assemblies (CCM-MEAs) in an applicable cell design, showing that the OER on iridium-based CCM-MEAs involves a concerted redox reaction of both the noble metal and oxygen, with the formation of active species occurring from mixed iridium oxo-hydroxides and crystalline IrO_2_ via oxidative charging of the catalyst.

## Introduction

Iridium oxide is the most promising oxygen evolution reaction (OER) catalyst for the industrial application of PEMWE, as it exhibits low overpotentials for the OER while possessing high stability even in acidic conditions^1^. It has been reported that amorphous forms of iridium oxide yield higher activities than the rutile IrO_2_ due to higher structural flexibility and lower redox stability^2,3^. Amorphous iridium oxides (IrO_x_) exist in varying compositions and properties depending on the synthesis route^4^. Furthermore, it has been commonly reported that iridium oxide catalysts can change composition with applied electrochemical bias harboring various iridium oxidation states and structural features, where amorphous IrO_x_ and crystalline IrO_2_ are known to transform into each other^5–8^. Formation of an amorphous hydrous oxide layer on IrO_2_ is occurring under OER conditions, as IrO_2_ surfaces are reported to be thermodynamically unstable under elevated potentials. Contrary to this, a number of studies have reported the formation of IrO_2_ at elevated potentials^9–18^. Previous ex situ Raman analyses on proton exchange membrane (PEM) electrolysis membrane electrode assemblies (MEAs) that were operated for several thousand hours exhibited clear IrO_2_ signals, contrary to the pristine samples exhibiting the Raman signature of amorphous IrO_x_^19^. This apparent discrepancy may be reconciled by considering different timescales. While amorphization is observed under *in operando* conditions at short timescales, IrO_2_ formation likely occurs over extended operation due to an overall increase in the material's oxidation state, thus favoring condensation of IrO_2_. Furthermore, the formation of volatile IrO_3_ has been observed by mass spectrometry at high potentials, contributing to iridium dissolution during the OER^20^.

**Fig. 1:**
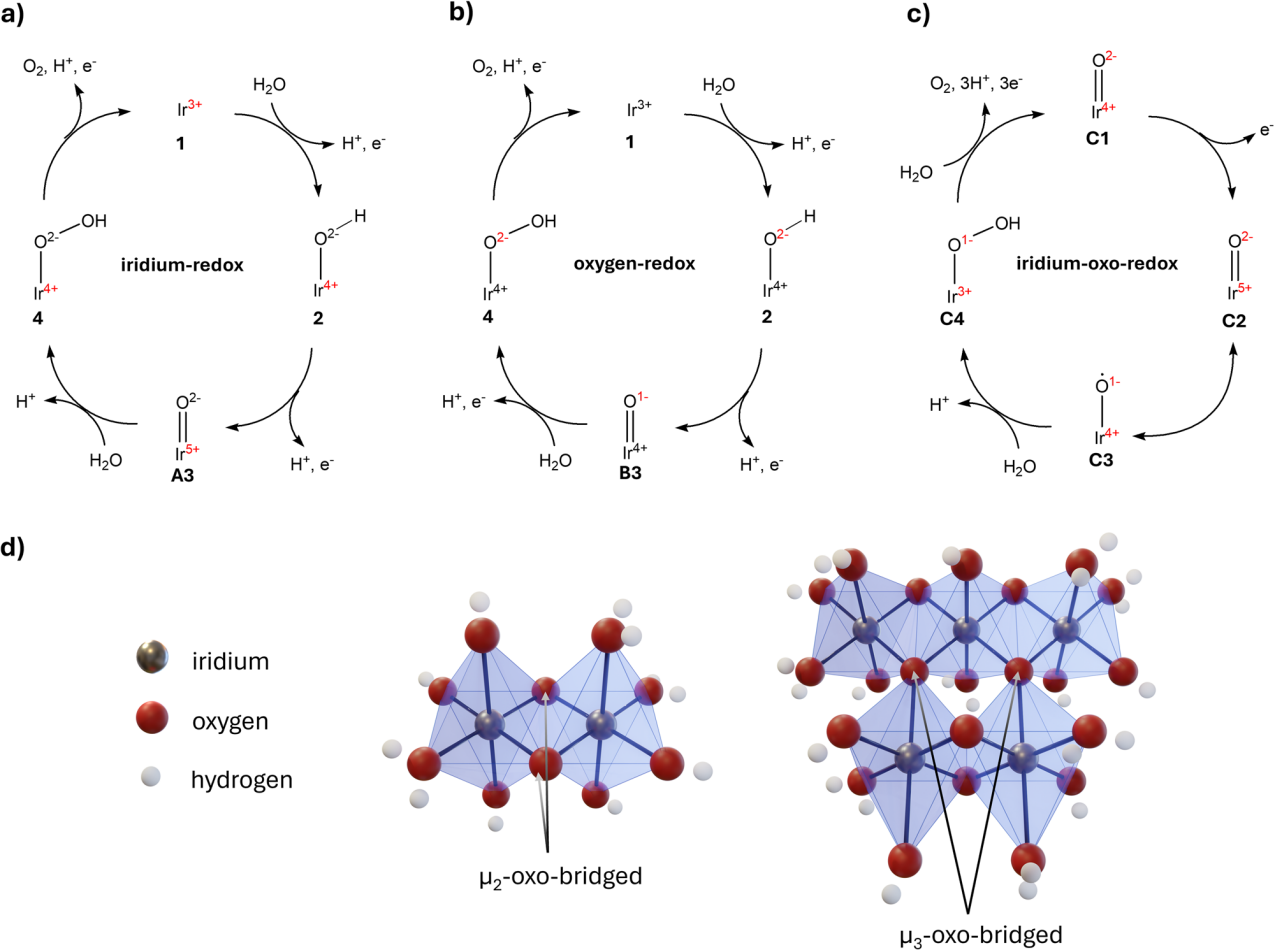
Three proposed catalytic cycles for the OER on iridium oxide and a structural model of IrOx. Three proposed catalytic cycles for the OER on iridium oxide and a structural model of IrO_x_. The “iridium-redox” cycle (**a**) is driven by the redox reaction of the iridium center^5,22–28^. The “oxygen- redox” cycle (**b**) is driven by the redox reaction of the adsorbed oxygen^5,22,25–32^. The “iridium-oxo- redox” cycle (**c**) is driven by the redox reaction of the Ir=O species^5,12,23,28,33^. Bridging oxygen ligands in IrO_x_ (**d**). Dimer of two iridium centers with octagonal coordination geometry connected by two μ_2-_oxo bridges and a further condensed form of IrO_x_ consisting of five iridium centers connected with μ_2_- and μ_3_-oxo bridging motifs.

While the OER at iridium oxides has been studied extensively, various discrepancies exist between the proposed catalytic cycles due to the different types of iridium oxides and electrolytes utilized in literature^21^. In the iridium-redox cycle seen in Fig. 1a, the reaction mechanism takes place via redox reaction of the iridium center, starting with the oxidation of Ir^3+^ (**1**) with H_2_O, forming an Ir^4+^-O^2-^-H species (**2**). The species is oxidized to Ir^5+^=O^2-^ (**A3**), subsequently forming an Ir^4+^-O^2-^-OH species (**4**), which relaxes to Ir^3+^ (**1**) under release of O_2_, closing the cycle^5,22–28^.

The oxygen-redox cycle, which is displayed in Fig. 1b distinguishes itself from the first one by the redox of the oxygen species instead of the iridium species. There, the Ir^4+^-O^2-^-H species (**2**) is oxidized to Ir^4+^=O^-1^ (**B3**) and the cycle proceeds *via* formation of the Ir^4+^-O^2-^-OH (**4**), like the first one^5,22,25–32^.

While the two previously discussed cycles are characterized by redox of either the iridium or oxygen, the iridium-oxo-redox cycle depicted in Fig. 1c exhibits the redox of the entire iridium-oxo state and was supported by previous in situ Raman studies on OER on iridium oxide. The cycle starts with the Ir^4+^=O species (**C1**) that gets oxidized to Ir^5+^=O (**A3**/**C2**), which is the precursor to an Ir^4+^-O˙^1-^ species (**C3**). Ir^3+^-O^1^-OH (**C4**) is then formed via proton transfer and releases O_2_, relaxing to Ir^4+^=O (**C1**) in the process^5,12,23,28,33^. The presence of iridium-oxo intermediates that define this catalytic cycle inherently involves deprotonation of the material prior to OER. Furthermore, a large part of the cycle, including water oxyl-coupling, proceeds chemically without electron transfer. These characteristics are in line with the observations of Nong et al., who demonstrated that the applied electrochemical bias does not directly act on the reaction coordinate. Instead, iridium-based electrocatalysts exhibit a pseudocapacitive behavior in which oxidative charge accumulation occurs via deprotonation, influencing bond formation/rupture chemistry by decreasing activation free energy of oxyl-water coupling linearly with the stored charge^34^. A recent study likewise identified electrophilic oxygen moieties as the OER-driving species and reported that, following an anodic pulse, switching to open circuit potential (OCP) resulted in greater oxygen release than stepping to a potential below OER onset. This observation suggests that once the active species are formed under applied bias, the subsequent oxygen evolution proceeds largely independently of the electrochemical driving force, consistent with a predominantly chemical reaction pathway^35^.

Bozal-Ginesta et al. identified three distinct redox transitions in IrOₓ and demonstrated that the highest-oxidized species, Ir^4.x+^, is the catalytically active state responsible for water oxidation, with its formation constituting the potential-determining step. Upon switching to open circuit, Ir^4.x+^ is spontaneously reduced back to lower oxidation states via chemical oxygen evolution, while lower-oxidized Ir^4+^ species are insufficient to drive water oxidation but can oxidize H₂O₂, suggesting that O–O bond formation is the rate-determining step and requires highly oxidized Ir centers. These findings are consistent with a mechanism in which the electrochemical bias serves primarily to generate the active high-valent species, while the subsequent oxygen evolution proceeds chemically^36^. In situ Raman analyses of anodically grown IrO_x_ at varying potential have been performed by Pavlovic et al.^13^. There, the system is described through oligomers of octagonally coordinated Ir^4+^ and Ir^3+^ atoms with hydroxide and water ligands. Di- and trimers of these IrO_6_ octahedrons were used for DFT calculations, with charges being balanced by the amount of water and hydroxide ligands. The amorphous IrO_x_ consists predominantly of oligomers formed by μ_2_-oxo bridging motifs as seen in Fig. 1d, where one oxygen ligand connects two iridium centers. IrO_2_ is formed by condensation of these edge-sharing [IrO_6_]_n_ polyhedra by the formation of μ_3_-oxo bridges. There, three iridium centers are connected by one oxygen bridge as seen in Fig. 1d, which is the bridging motif found in crystalline IrO_2_. Thus, IrO_2_ activation can be seen as the unraveling of the structure by hydration. The distinct oxygen coordination environments of amorphous and crystalline IrO_x_ are reflected in their XPS O 1*s* signatures. Roiron et al. demonstrated that the relative contributions of μ_1_-O(H), μ_2_-O(H), and μ_3_-O species serve as quantifiable descriptors of the amorphous versus rutile character of IrO_x_ materials, with purely amorphous phases exhibiting a higher proportion of defective, singly-coordinated μ_1_-O(H) species, while the μ_3_-O contribution, bridging three iridium centers, is present exclusively in crystalline materials^37^.

The description of the catalyst system in this work as molecular species, instead of a semi-crystalline solid, enabled the simulation of the IrO_x_(OH)_y_(H_2_O)_z_ clusters and their theoretical Raman spectra via DFT calculations. In situ electrochemical experiments in 0.5 M H_2_SO_4_ and H_2_O, as well as D_2_O and ^18^OH_2_, supported by the calculations, were used to assign the peaks to vibrational bands. An overview of the identified Raman signals, which were labeled with Greek letters by Pavlovic et al.^13^, is depicted in Fig. S1a. It was demonstrated by isotope-labeling experiments that peaks α and β are decoupled from O-H vibrations. Based on these findings, α and β were assigned to twisting vibrations of the oxo-bridges along the Ir-Ir axis. At least two kinds of Ir centers were suggested based on the behavior of peaks γ, δ, and ε at varying potentials. A redshift of peaks γ and ε happening in a correlated manner during a potential sweep is evident from the measurements. Based on these findings and simulation of Ir^4+^ oxohydroxides, γ and ε were assigned to stretching vibrations of the μ_2_-oxo bridges involving Ir^4+^ sites only. Structure optimization of Ir^3+^/Ir^4+^ di- and trimers resulted in protonation of μ_2_-bridging oxygens. Based on the similarity to simulated Raman spectra of these compounds, peak δ was assigned to the Ir^3+^ site due to its disappearance with increasing potential between 0.8 and 1.2 V (vs. RHE). Further increase of the potential led to halting of the redshift of γ and ε as well as appearance of the E_g_ band of rutile IrO_2_, but no further changes in the IrO_x_ structure. The start of the OER correlated with the stop of the red shift of γ and ε. The absence of further structural change led to the conclusion that the red shift is caused by overall charging of the structure until the oxidative pressure is released by water oxidation upon start of the OER^13^.

Surface enhanced Raman spectroscopy (SERS) measurements of electrodeposited IrO_x_ in alkaline electrolyte were performed by the same group, revealing an additional η peak (813 cm^−1^) at higher potentials. This signal was assigned to the OER active Ir=O moiety. In acidic media, the η peak is significantly less intense, as the structure is much more condensed, resulting in less exposed active sites^33^. In order to exclude the possibility of Au oxide formation in SERS measurements on an Au substrate, Saeed et al. utilized shell-isolated nanoparticle enhanced Raman spectroscopy (SHINERS)^12^. Instead of a nanostructured gold substrate generating the surface enhancement, SiO_2_-coated Au nanoparticles were drop-cast onto the electrodeposited IrO_x_. With this method, they were able to measure Raman spectra while sweeping to higher potentials at a rate of 5 mVs^−1^. Contrary to the results of Pavlovic et al., they observed a strong potential dependence of the α and β peaks, assigning them to Ir-O-Ir twisting vibrations involving Ir^4+^ or Ir^4.x+^, respectively. Furthermore, three peaks were assigned to the Ir-O-Ir stretching vibration, which changes position with increasing potential as depicted in Fig. S1b. At low potentials from open circuit potential (OCP) to 0.5 V vs. Ag/Ag^+^, this vibration manifests as peak ε at 719 cm^−^^1^. At increased potentials between 0.6 V – 1.1 V vs. Ag/Ag^+^, the same vibration is visible as peak ζ at 773 cm^−1^ and with further increased potential between 1.2 V and 1.8 V vs. Ag/Ag^+^, it is located at 672 cm^−1^ designated as peak θ. While the shift of ε to θ was already explained by Pavlovic et al. to be caused by charging of the structure, it is left open by the authors in this study why the shift to ζ occurs in between. Furthermore, signal ζ has not been observed in any of the other related studies^13,33^.

*In operando* studies on IrO_x_ OER catalysts, mostly academically relevant samples with ideal materials like electrodeposited or anodically grown IrO_x_. To our knowledge, no industrially relevant membrane electrode assemblies have been utilized, which differ by the use of perfluorinated sulfonic acid (PFSA) polymer membranes as the electrolyte, as well as significantly less defined iridium catalyst compositions and structures. In this work, the applicability of Raman spectroscopy as an analysis tool was demonstrated by performing in operando Raman measurements of a commercial PEMWE MEA at various cell potentials. The models utilized in previous Raman studies were implemented for the deconvolution of the spectra and adapted for the interpretation of the investigated system.

## Results

### Catalyst evolution during electrolysis

**Fig. 2:**
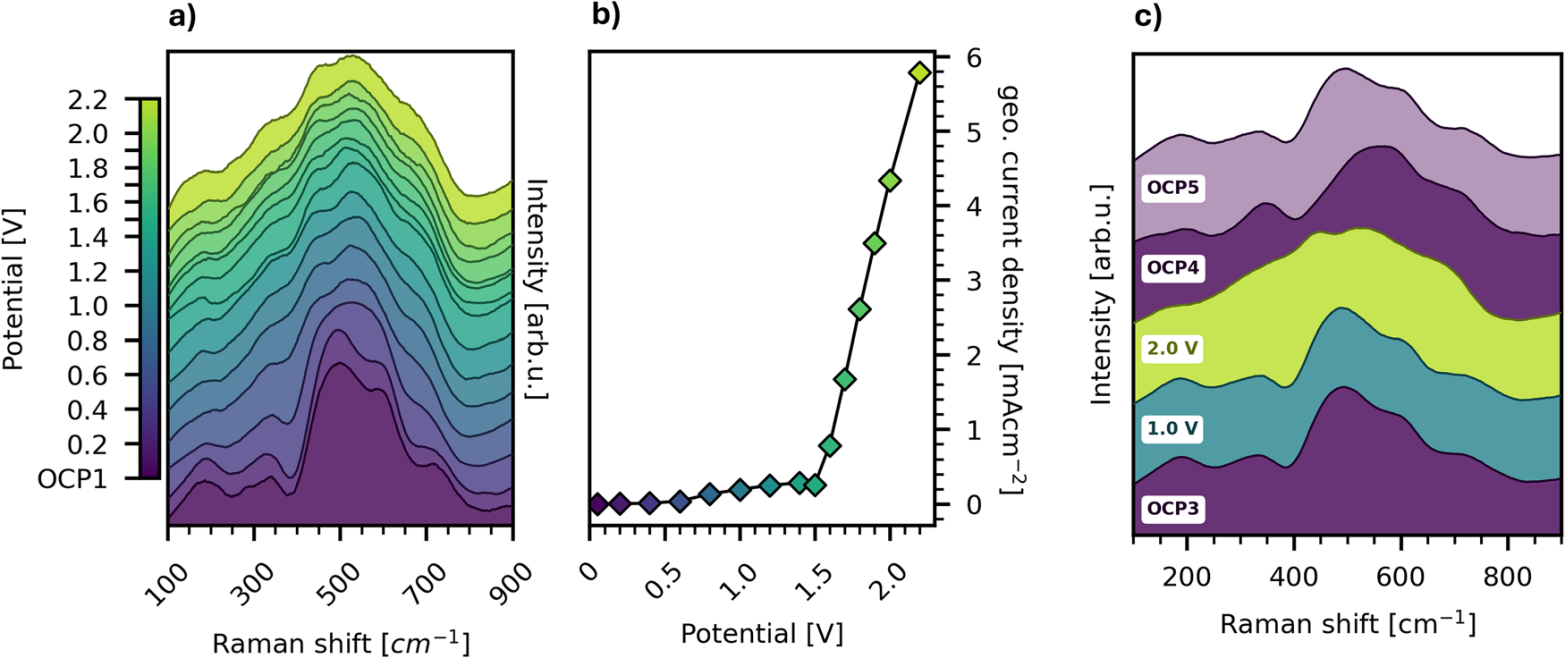
Results of the *in operando* experiment. Raman potential series of an iridium oxide-based commercial PEM anode (**a**). Geometrical current density at the applied potential (**b**). The current density was calculated from the last measured current for each potential. Raman spectra during stages 4–6 (**c**).

Stages 1 and 2 (see “Methods”/Fig. S4) were performed to evaluate the potential dependent evolution of the electrocatalyst's structure. Here, the open circuit potential OCP1 was performed in stage 1, followed by chronoamperometry (CA) measurements with increasing potentials in stage 2. Figure 2a depicts the Raman spectra of the IrO_x_ OER catalyst as a function of applied cell potential. At OCP1, the IrO_x_ Raman signature is characterized by an intense main feature between 400 and 800 cm^−1^ and two smaller bands at approximately 180 and 310 cm^−1^. The main feature itself consists of three features centered at 500, 600, and 710 cm^−^^1^. The main feature broadens considerably at a cell potential of 0.4 V, resulting in significant overlap between the spectral features, which were observable as distinct shoulders at 500 and 600 cm^−1^ up to this point. This broadening causes a significant intensity increase at 370 cm^−1^. Furthermore, the shoulder located at 710 cm^−1^ is redshifting at this cell potential, merging further into the main feature.

This trend continues at 0.6 V, finally resulting in a very broad feature with multiple shoulders, but no distinct components at a cell potential of 0.8 V. No significant changes occur with further increase of the cell potential, until 1.4 V, where the left tail of the feature between 200 and 400 cm^−1^ decreases significantly in intensity, which, however, increases to previous levels at 1.5 V. The spectrum is constant until the maximum applied potential of 2.2 V.

The geometrical current density as a function of cell potential is depicted in Fig. 2b. As indicated by the significant current response starting between 1.5 and 1.6 V, the start of the OER is evident. The changes in the Raman spectra correlate with the current increase between 0.4 and 1.5 V, and no further changes occur as soon as the OER starts.

The potential dependent Raman signature overall matches the experiments of Pavlovic and Saeed, however, with slight deviations^12,13,33^. In alkaline conditions, signal η occurs at 813 cm^−1^ during the OER and scales with the anodic current. Due to the acidic conditions in PEMWE, this signal is absent. Saeed et al. observed an additional signal ζ at 773 cm^−1^ occurring concertedly with α, which was assigned to a blue-shifted Ir^4+^-O-Ir^4+^ stretching vibration^12^. This signal is also absent in the recorded *in operando* spectra. The ζ signal may be associated with surface species inaccessible in acidic conditions or may be only stable over the short time scale in which the SHINERS measurements were performed. It is also plausible that enhanced sensitivity due to the use of SHINERS enabled the observation of this signal. Furthermore, it is evident that the changes in the spectra are mostly completed at significantly lower potentials than the OER onset. In the previous studies, significant changes occurred up until the OER^12,13,33^.

### Catalyst relaxation

Stages 3 and 4 were performed to reset the catalyst’s state after the potential series in stage 2 in preparation for the next experiments. Both consist of a cyclic voltammetry (CV) experiment followed by an OCP experiment (see “Methods”/Fig. S4). Potential cycling has been reported to reactivate IrO_x_ catalysts by hydration and reduction of condensed IrO_2_ phases^14,38,39^. Cycling to 0.0 V at the end of the CV1 and CV2 is done to reduce higher iridium oxidation states. The OCP experiments after the CVs were performed to allow the catalyst to relax into an equilibrium state before continuing with stage 5. The Raman spectra of the OCP steps are displayed in Fig. S10. At OCP2 after the first cycling step, an overall shift of the IrO_x_ main feature to higher wavenumbers is visible compared to the pristine sample, suggesting an increased ratio of Ir^3+^ species. At OCP3, following the second potential cycling step, the Raman spectrum closely resembles that of the pristine catalyst, indicating that the starting conditions of stage 5 are sufficiently comparable to those of stage 1.

After resetting the catalyst, further experiments were performed to observe the relaxation of the catalyst after the OER. The respective Raman spectra recorded in stages 4–6 are displayed in Fig. 2c. During OCP3, the Raman spectrum is comparable to the catalyst's initial state in stage 1, indicating that it is reset to its pre-OER state by the combination of potential cycling and OCP. Stage 5 started with the application of 1.0 V cell potential for 50 min, resulting in no significant change in the spectra at a current density of 0.016 mA cm^−2^. Increase of the cell potential to 2.0 V for 60 min resulted in a significant electrolysis current density of 4.4 mA cm^−2^ and similar changes in the Raman spectra as already observed in the previous potential series. The electrochemical bias was removed with the start of OCP4, allowing relaxation of the catalyst. After 15 h of OCP4, the Raman spectrum exhibits a blueshift of the center of mass for the broad IrO_x_ band. Additionally, the peak at 310 cm^−1^ is significantly more pronounced compared to OCP3 in stage 4 and the CA measurements in stage 5. After an additional 24 h of OCP in stage 6, the Raman spectrum returned close to the initial state.

### Deconvolution of the *in operando* Raman spectra

In order to study the evolution of each component of the Raman spectra, various fitting models were derived based on peak assignments from the Raman studies mentioned earlier^12,13,33^ and implemented for deconvolution of the spectra utilizing a custom Python script. The fitting procedure was performed 10 times with slight random variations of the fitting parameters for each spectrum in order to evaluate the stability of the fitting model. The exact fitting procedure is described in the methods section.

**Fig. 3:**
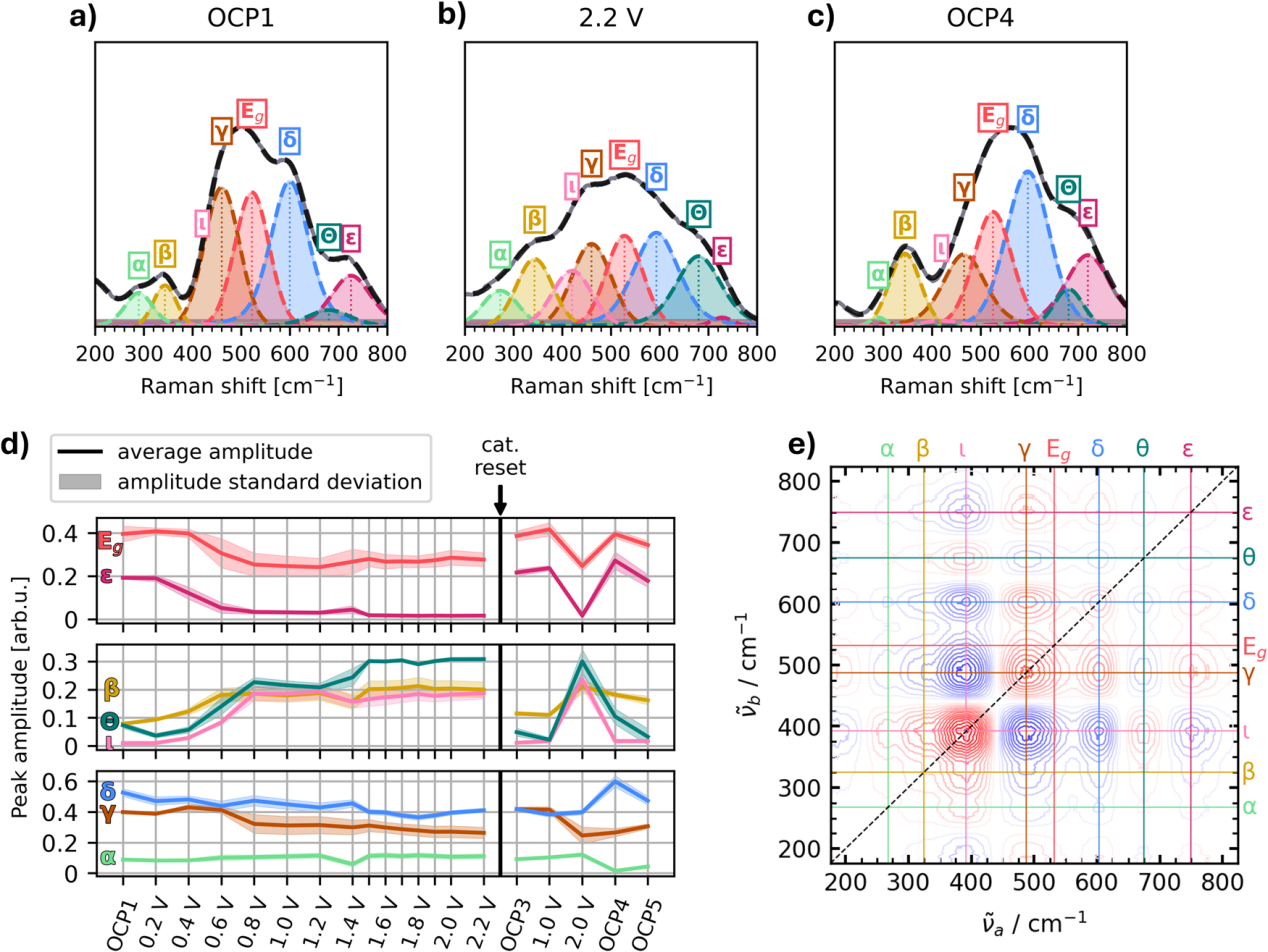
Spectral analysis of the in operando experiment. Spectral analysis of the *in operando* experiment. Fits of Raman spectra at OCP1 (before potential series) (**a**) at 2.2 V (end of potential series) (**b**), and at OCP4 (during catalyst relaxation) (**c**). Fitting was done using a model consisting of 7 Pseudo-Voigt peaks for IrO_x_-related signals, as well as one Pseudo-Voigt peak for the IrO_2_ E_g_ band. Peak amplitudes from deconvolution of the spectra for the respective applied potentials and OCPs (**d**). Peaks related to IrO_2_ (top); Peaks assigned to species with higher oxidative charge (middle); Peaks assigned to Ir^4+^-O-Ir^4+^ and Ir^4+^-O-Ir^3+^ vibrations (bottom). Values are averaged over 10 runs of the fitting procedure with randomly varied starting parameters. Synchronous correlation spectrum of stages 1–2 of the *in operando* series (**e**). Positive values are depicted in red, negative values are depicted in blue. Peak positions as determined from the synchronous correlation spectrum are depicted using colored vertical and horizontal lines. Fits of all spectra are depicted in Fig. S14.

The fitting model was developed iteratively, based on and expanded upon previous works described in the introduction. In particular, the developed model takes into account the E_g_ band of crystalline IrO_2_ and two additional peaks to reflect the spectral changes through variation of peak amplitudes instead of shifting the peak positions of γ and ε. The detailed process of development of the fitting model is described in the supplementary information. The final model considers 7 peaks for the IrO_x_ Raman signature and one peak representing the E_g_ band of IrO_2_. Representative spectra and their respective fits are displayed in Fig. 3a–c.

As illustrated in Fig. 3d, the E_g_ band amplitude drops upon potential increase between 0.2 and 0.8 V, indicating an amorphization of the electrocatalyst as the OER potential is approached. Additionally, it is evident that the evolution of the ε amplitude is correlated with E_g_. These findings indicate that the ε band could be related to IrO_2_ instead of amorphous IrO_x_. This assumption is supported by the fact that the Raman shift of the ε band matches that of a convoluted peak of broadened and overlapping IrO_2_ A_1g_ and B_2g_ bands. Furthermore, the bimodal formation of peaks evident from the DFT calculations of the Ir^4+^ trimer performed by Pavlovic et al. is significantly more narrowly distributed than the experimentally observed signals γ and ε^13^. The lower shift of the experimentally observed E_g_ band compared to literature^40^ can be understood by the implementation of IrO_2_ crystals in an amorphous IrO_x_ matrix, which may affect the force constants of the IrO_2_ vibrations due to mechanical influences^41,42^. Bands β, θ, and ι are characterized by their increasing amplitudes with rising potential up to the start of the OER. While the concerted appearance of bands β and θ has been reported by Saeed et al.^12^, the additional ι peak exhibits a very similar trend, indicating that they are related to the same species. Based on the previous literature and the increase in amplitude, increasing potential up to the OER, these peaks are assigned to charged IrO_x_ structures. After OER onset at 1.5 V, β, θ, and ι generally remain at constant values. The δ band decreases gradually over the potential increase up to 2.2 V, while γ exhibits an increase of about 10% in amplitude for the potential range from 0.2 to 0.4 V, followed by a gradual decrease comparable to the δ band. This indicates that both species are consumed for the formation of species associated with the β, θ, and ι bands up to the OER onset. Therefore, signal δ and γ are assigned to Ir^3+^ and Ir^4+^ species, respectively, which is in line with literature^12,13,33^. Component α does not exhibit significant potential-dependent behavior. A prominent change in peak amplitudes is observed at 1.4 V, where the spectrum exhibits anomalously reduced intensities below 500 cm^−1^ compared to adjacent potentials. This is attributed to a shift in optical parameters at the onset of gas bubble formation rather than a meaningful change in catalyst structure. As the fitting procedure uses parameters from each spectrum as starting values for the next, this local deviation propagates into subsequent fits, as evidenced by the elevated standard deviation of the θ amplitude at higher potentials. The apparent increase in θ amplitude is therefore considered a fitting artifact and is not interpreted as a change in catalyst oxidation state.

In stages 4–5, no significant changes in peak amplitudes are observed upon increasing the potential to 1.0 V from OCP. This contrasts with the behavior observed in stage 1, where significant spectral changes occurred below 0.8 V, and is likely attributed to the shorter and less gradual potential protocol, which does not allow sufficient time for the catalyst to structurally adapt to the applied bias. An amplitude decrease for the E_g_ and ε bands is observed upon stepping to 2.0 V from OCP3, similar to the behavior in stages 1–2. In stage 6, 15 h after the start of OCP4, both components revert to their respective amplitudes at OCP1. The β, θ, and ι bands exhibit a similar trend to that seen in stage 2. This is further supported by the XRD data of the pristine and operated samples, displaying no significant differences in the iridium oxide reflexes (Fig. S6).

**Fig. 4:**
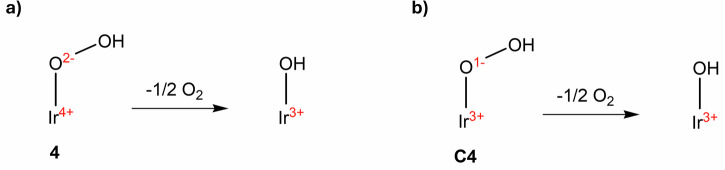
Decomposition of iridium-hydroperoxo species into Ir^3+^. Decomposition of iridium-hydroperoxo species into Ir^3+^. Decomposition of species 4 in the iridium-redox and oxygen-redox cycles (**a**); Decomposition of species C4 in the iridium-oxo-redox cycle (**b**).

A prominent evolution occurs for the δ band, which exhibits a significant spike in amplitude at OCP4 in stage 6, even surpassing its initial value in stage 1. The open circuit conditions after shutdown of the cell might facilitate the decomposition of the hydroperoxo-species (**4**/**C4**, Fig. 1) without electron transfer. This might result in the formation of an Ir^3+^-OH species as depicted in Fig. 4, explaining the high amplitude of δ at OCP4.

The γ band intensity drops by almost 50% with the increase from 1.0 to 2.0 V and only slowly regains it over the next 40 h in OCP4 and OCP5. It is evident that the electrocatalyst exhibited lower Ir^3+^ concentrations at OCP3 in stage 4 compared to stage 1. The lower Ir^3+^ starting concentration potentially results in slower Ir^4+^ formation by oxidation of Ir^3+^ during the potential increase in stage 5. In turn, the formation of Ir^4+^ is too slow to compensate for the higher reaction rate of Ir^4.x+^ formation from Ir^4+^ at elevated potentials, resulting in the low amplitude of the γ signal at 2.0 V. Generally, at OCP5, all amplitudes approach the values found for the pristine catalyst at OCP1, indicating that the transformations of the catalyst are reversible.

Supplementary to this fitting procedure, two-dimensional correlation analysis was performed with the data of stages 1 and 2. The method, which was pioneered by Noda^43^, allows for correlational analysis of dynamic spectra of a system under any type of perturbation. In this case, an electrochemical perturbation is employed, resulting in dynamic, potential-dependent Raman spectra. The correlational analysis results in a visual representation of the correlation between two spectral positions under perturbation, essentially deconvoluting spectral signatures consisting of correlating overlapping signals. Compared to the previously discussed fitting procedure, this is a model-free analysis approach, free from human bias.

Utilizing Eqs 2 and 3 (see “Methods”), the correlation spectra were computed for stages 1 and 2 of the *in operando* Raman series. In the synchronous spectrum (Fig. 3e), changes in intensity occurring synchronously between two peaks along the potential axis are shown. Positive values are the result of two peaks which increase or decrease concertedly with the potential, and negative values occur if one peak increases while the other one decreases in intensity. The diagonal peaks naturally exhibit positive values as they show the correlation of one peak with itself. Overall, diagonal and cross peaks are visible in the synchronous spectrum at Raman shifts close to the positions of the peaks that were used for the expanded fitting procedure. Peaks α and β show up as shoulders of the ι peak, and the E_g_ peak shows as a shoulder of the γ peak. The intensity changes of the peaks with potential are also reflected very well in the synchronous spectrum. Peaks β, θ, and ι show positive cross peaks with each other and negative cross peaks with peaks γ, E_g_, δ, and ε, and vice versa. This reflects the observation from the utilized fitting model where β, θ, and ι, and γ, E_g_, δ, and ε demonstrate the same correlated behavior. Overall, the results of the two-dimensional correlation analysis strongly support the implementation of eight peaks for the fitting model and their Raman shifts.

## Discussion

**Fig. 5:**
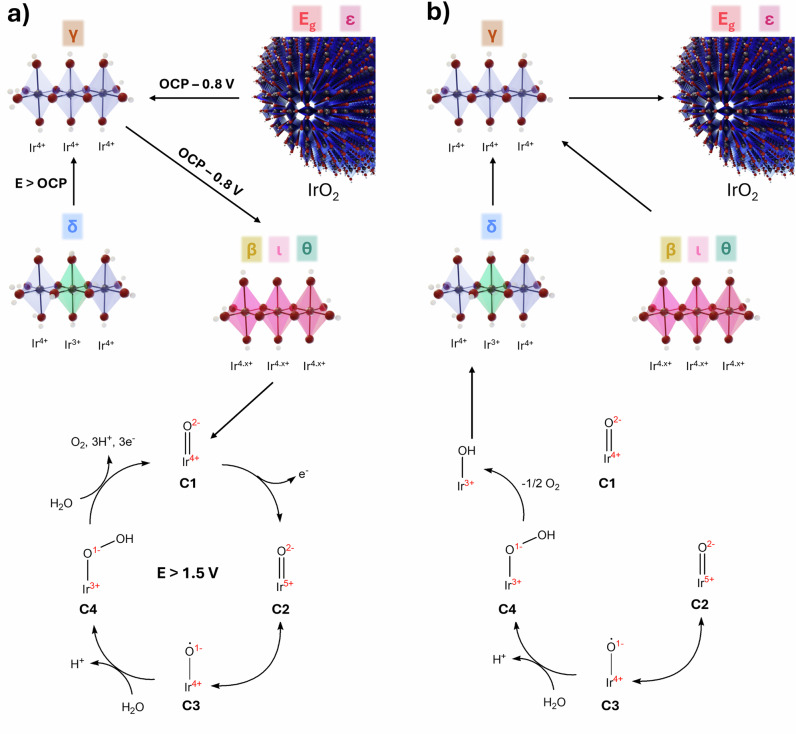
Proposed mechanism of the observed structural changes. During potential increase (stage 1–2) (**a**). After shutdown (stage 6) (**b**). Potential ranges in which the processes take place are noted above the respective arrows. Corresponding Raman signals of the IrO_x_ species are depicted above the structures.

A model explaining the observable changes in the Raman spectra was derived based on the results of the potential series and the catalyst relaxation. The mechanism taking place during the potential series is depicted in Fig. 5a. The evolution of Raman bands evident from the deconvolution of spectra provides evidence for a significant change of the catalyst associated with the formation of a charged pre-catalytic species before the start of the OER. Oxidation of Ir^3+^ to Ir^4+^ occurs, as derived from the concurrent decrease of the δ band and increase of the γ band between OCP and 0.4 V. The steady decrease of the δ and γ bands over the rest of the potential range is a manifestation of the transformation of Ir^3+^ and Ir^4+^ species into charged species. Also, with increasing potential from OCP to 0.8 V, crystalline IrO_2_ is amorphized into hydrous IrO_x_, as indicated by the decrease in peaks E_g_ and ε. In the same range, charged Ir^4.x+^ species are formed, resulting in the increase in signals β, θ, and ι. Thus, a more oxidized, amorphous form of iridium oxide is generated before the OER, which is in line with the iridium-oxo-redox cycle and the mechanism based on catalyst charging proposed by Nong et al^5,12,23,28,33,34^.

Contrary to previous Raman studies, the changes in the Raman signature are largely completed at 0.8 V. This behavior could be rooted in the integration of the catalyst in the ionomer in PEMWE MEAs. The adjacent ionomer accelerates charge accumulation in the catalyst structure via deprotonation of iridium oxo-hydroxides by facilitating proton conduction away from the anode. For OER catalysts without a proton exchange ionomer, proton transport will take place at a higher potential due to the lack of a strong acid. This interpretation is thus in line with previous Raman studies, where similar spectral changes are occurring up until the OER onset^12,13,33^. Further oxidation to form the active iridium-oxo species (C2) then occurs at potentials above 1.5 V, resulting in the observed OER current. The iridium-oxo species is hardly observable under acidic conditions, and the iridium-hydroperoxo species has a similar Raman signature as the Ir^4+^ containing structures, explaining the lack of change in the Raman spectra at OER onset in the analyzed PEMWE system^12,13,33^.

The mechanism explaining the changes in Raman signature during catalyst relaxation is depicted in Fig. 5b. The significant increase of δ, i.e., the amplified formation of Ir^3+^ after shutdown of the cell, hence without current flow, strongly supports the claim that the OER proceeds largely chemically instead of electrochemically. The formation of Ir^3+^ indicates quenching of the active oxidatively charged catalyst through redox reactions. This likely occurs by means of water oxidation without electron transfer via the pathway from **C2** to **C4** proposed in the iridium-oxo-redox cycle and consequential decomposition of the unstable Ir^3+^-O^1^-OH species, as illustrated in Fig. 4. After extended time at OCP, the catalyst’s structure reverses back to its pristine state, which is a mixed iridium oxide.

In conclusion, the developed experimental setup and fitting procedure enabled the in-depth *operando* analysis of OER catalysts inside an operating PEMWE electrolyzer cell. The thereby assessable evolution of peak amplitudes gave insight into the nature of the OER mechanism in iridium-based PEMWE OER electrocatalysts that exist within a proton exchange ionomer network. With increasing potential, the amorphization of IrO_2_ and the accumulation of oxidative charge in the IrO_x_ catalyst could be monitored prior to the OER onset. The hereby presented study suggests a distinct role of the proton-conducting ionomer in application-oriented MEAs, as it may promote oxidative charging by facilitating proton removal from the anode, potentially contributing to the substantial structural changes observed well before the OER onset. Finally, the generation of increased concentrations of Ir^3+^ during current-less catalyst relaxation was demonstrated. These findings are evidence that the OER takes place on an oxidatively charged catalyst with active iridium-oxo sites and exhibits a strong chemical rather than an electrochemical character.

## Methods

### Electrochemistry—*in operando* Raman

Electrochemical experiments were conducted on a Gamry reference 3000 potentiostat with an in-house designed electrolysis Raman cell (Figs. S2 and S3) and a commercially available PEMWE MEA (Ion Power GmbH, Germany). The MEA consists of a Nafion N115 membrane with an anode Ir (IrO_x_) loading of 1.0 mg cm^−2^ and a cathode Pt (Pt/C) loading of 0.3 mg cm^−2^. The cell consists of two 3D printed PETG parts with cutouts to ensure mass transport to and from the electrodes. Pt-coated Ti meshes with a wire thickness of 0.3 mm and a hole size of 1.14 mm, resulting in a mesh fineness of 17.6 mesh, are used for contacting the electrodes. The meshes are mounted to the top and bottom parts of the cell using brackets. The top part of the cell features hose connections, allowing for water circulation via 4.8 mm diameter silicone tubing. Water was pumped with a Watson–Marlow 530 peristaltic pump (Watson-Marlow GmbH, Germany). A technical drawing with the relevant dimensions of the cell is depicted in Fig. S3. The whole cell is mounted in a PTFE vessel filled with approximately 50 ml of milliQ water.

The *operando* experiment is divided into six stages. The detailed sequence of electrochemical experiments is depicted in Fig. S4. The first stage began with OCP for 2 h. Subsequently, chronoamperometry (CA) experiments were conducted in stage 2, applying the constant cell potentials between 0.2 and 2.2 V for at least 20 min. The detailed voltage profile and current response are depicted in Fig. S5. Afterwards, stages 3 and 4 follow, each starting with a cyclovoltagram (CV) between 0.0 and 3.0 V with 20 mVs^−1^ for 4 cycles, cycling back to 0.0 V at the end of the CV. Each CV was followed by OCP measurements. CV1 and 2 are displayed in the supplementary information (Fig. S7). In stage 5, a constant cell potential of 1.0 V was applied for 50 min, followed by 2.0 V for 1 h. Subsequently, the setup was held at OCP for 39 h total in stage 6. During operation, water was circulated around the objective to prevent gas bubbles from getting stuck on the objective lens.

### Raman experiments

Raman experiments were performed using a WITec/Oxford Instruments (Ulm, Germany) Alpha 300 R confocal Raman microscope. A 633 nm Laser and a Zeiss W Plan-Apochromat 63x/1.0 M27 water immersion objective were utilized. For detection of the Raman signal, a WITec/Oxford Instruments UHTS 300S spectrometer with a 600 g/mm grating and a back illuminated deep depletion CCD camera was used. Spectra were measured as point measurements with one accumulation for 120 s of integration time. Laser power is measured using the WITec True Power device and corrected by accounting for power losses at optical components along the whole beam path. Thus, laser power reaching the sample surface can be determined.

The *in operando* measurements were performed at a laser power of 0.16 mW, losses through the beam path were determined to be 37.5%, and the objective transmittance at 633 nm is around 90%, resulting in an effective laser power of 0.09 mW at the sample surface. Raman time series experiments were initiated only after the initial current drop had settled to approximately stable values, each acquiring 10 spectra per potential. The spectra were corrected for cosmic rays, and the average spectra were computed for every time series. A linear background was utilized for background subtraction. The spectra were then normalized to the peak area between 100 and 800 cm^−1^ and post-processed using a Savitzky–Golay filter with a polynomial order of 2 and a window size of 40.

### Fitting procedure

The fitting procedure was scripted utilizing the Lmfit 1.3.2 module^44^ in Python. The Levenberg–Marquardt algorithm was used for fitting with a maximum number of iterations of 2000·(*n* + 1), where *n* is the number of variable parameters in the model. A pseudo-Voigt function was utilized to implement the peaks in all discussed models, as seen in Eq. 1.

1 $$\begin{array}{c}f\left(x;A,{x}_{{{\mathrm{cen}}}},\sigma ,\alpha \right)=\frac{\left(1-\alpha \right)A}{{\sigma }_{g}\sqrt{2\pi }}{e}^{[-{\left(x-{x}_{{{\mathrm{cen}}}}\right)}^{2}/2{\sigma }_{g}^{2}]}+\frac{\alpha A}{\pi }\left[\frac{\sigma }{{(x-{x}_{{{\mathrm{cen}}}})}^{2}+{\sigma }^{2}}\right]\\ {\sigma }_{g}=\,\sigma /\sqrt{2{{\mathrm{ln}}}2}\end{array}$$

x: Independent variable, A: Amplitude, ${x}_{{{\mathrm{cen}}}}$: Center, σ: Characteristic width, ${\alpha }_{{frac}}$: Fraction (relative weight of Gaussian and Lorentzian components).

The termination criteria, namely the desired relative errors in the approximate solution and in the sum of squares, as well as the desired orthogonality between the function vector and the Jacobian matrix, were set to 10^−14^. The Raman spectra for increasing cell potentials were fitted successively, where the fit results of the previous spectrum are utilized as starting values for the following spectra. Additionally, limits to the variation of components of two consecutive spectra were implemented, thus assuming a gradual change of the peaks with a gradually changing potential. Random variations for A, μ, σ, and α are applied to the initial fitting values for the Pseudo–Voigt peaks (Eq. 1). The random variations lay in the range of -−0.1 to 0.1 for A and α, −2 to 2 μ and −1 to 1 for σ. This ensures that first, the fitting algorithm does not converge towards a local minimum, and second, the stability of the fitting model can be evaluated. For the evaluation of stability, each fitting model was run 10 times with random variations of initial fitting parameters, averaged peak amplitudes and standard deviations were calculated for each component. In case of an unstable fitting model, a high run-to-run variance and thus a high standard deviation is expected.

### Two-dimensional correlation analysis

Two-dimensional correlation analysis was performed with the Raman data from stages 1 and 2. The synchronous correlation spectrum was calculated as follows:

2 $$\begin{array}{c}\widetilde{y}\left(\widetilde{\nu },\,E\right)=\left\{\begin{array}{c}y\left(\widetilde{\nu },\,E\right)-\,\bar{y}\left(\widetilde{\nu }\right){for}\,{E}_{\min }\le E\,\le \,{E}_{\max }\\ 0\,\,{otherwise}\end{array}\right.\\ \bar{y}\left(\widetilde{\nu }\right)=y\left(\widetilde{\nu },\,{OCV}\right)\end{array}$$

E: Cell potential, $\widetilde{y}$: Dynamic spectrum, y: Spectrum, $\bar{y}$: Reference spectrum, ${E}_{\min }$: Lowest potential, ${E}_{\max }$: Highest potential

3 $$\varPhi \left({\widetilde{\nu }}_{a},{\widetilde{\nu }}_{b}\right)=\frac{1}{m-1}{\widetilde{y}\left({\widetilde{\nu }}_{a},\right)}^{T}\widetilde{y}\left({\widetilde{\nu }}_{b}\right)$$

Φ: Synchronous correlation spectrum, ${\widetilde{\nu }}_{a},{\widetilde{\nu }}_{b}$: Spectral positions

A more detailed description of the method is depicted in the supplementary information.

## Supplementary information

Supplementary Information

## Data Availability

The electrochemical and operando Raman spectroscopy data are available in the openly accessible Jülich Data repository at 10.26165/JUELICH-DATA/8F356Q.

## Code availability

The spectral fitting and plotting scripts used in this study are available in the openly accessible Jülich Data repository at 10.26165/JUELICH-DATA/8F356Q. The fitting scripts were developed using Python v3.11.5 with the lmfit module v1.3.2, the numpy module v2.3.5, and the rampy module v0.6.4.
