# Supplementary material for: Insights into the Ir-O redox dynamics of an operating PEM water electrolyzer using in operando Raman spectroscopy
Source: Commun Chem. 2026 Aug 6;9:270. doi: 10.1038/s42004-026-02162-9 (PMC13447846; doi:10.1038/s42004-026-02162-9)
Supplement: Supplementary file 1 [file 42004_2026_2162_MOESM1_ESM.pdf]

Supplementary - Insights into the Ir-O redox dynamics of an operating PEM water electrolyzer using in operando Raman spectroscopy

Raman peak positions of IrO_x in literature

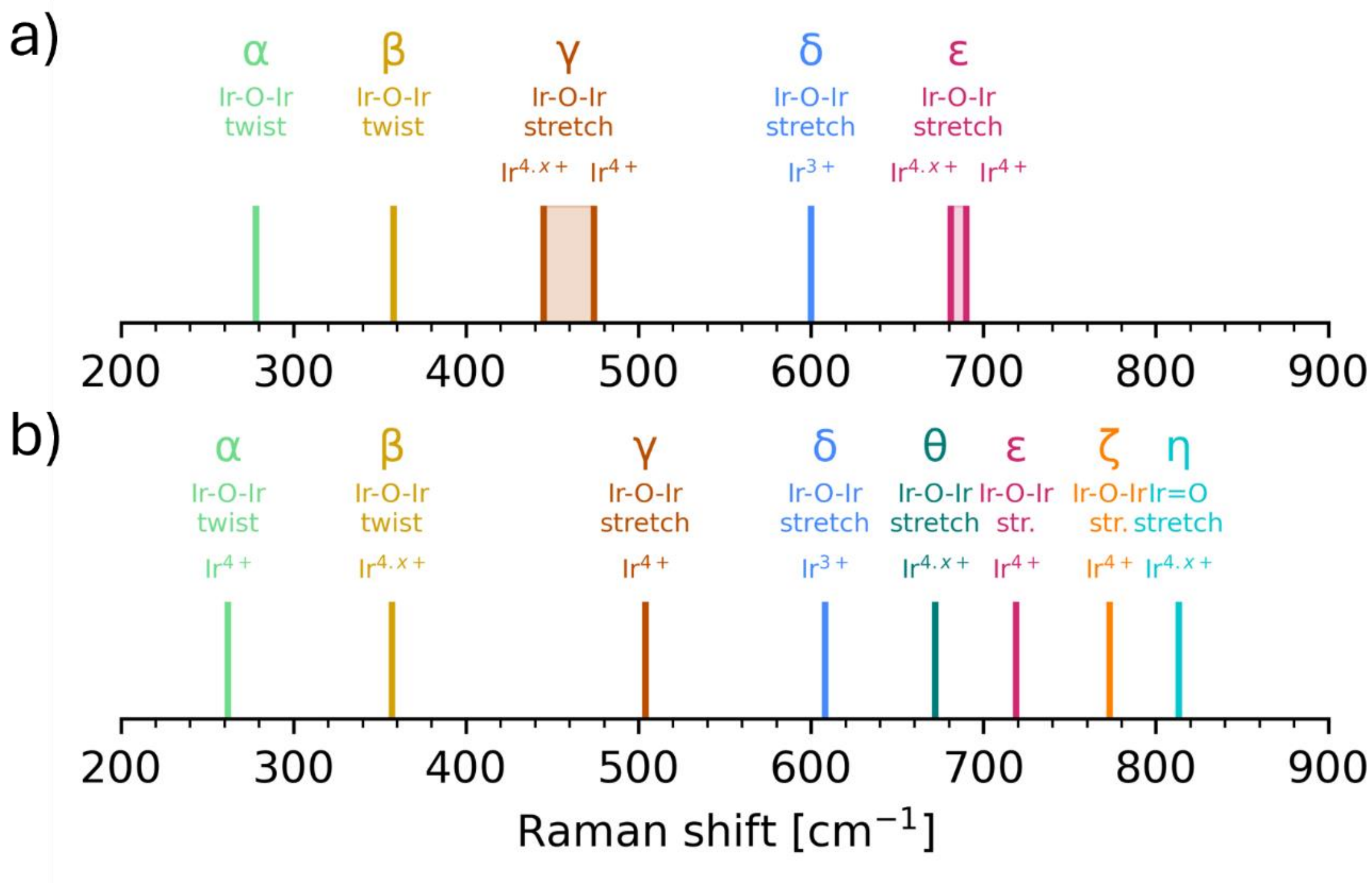

Fig. S1: Overview of identified peaks in the *in situ* analysis performed by Pavlovic et al.[1] (a); Overview of identified peaks in the *in situ* analysis performed by Saeed et al.[2] (b)

Cell construction

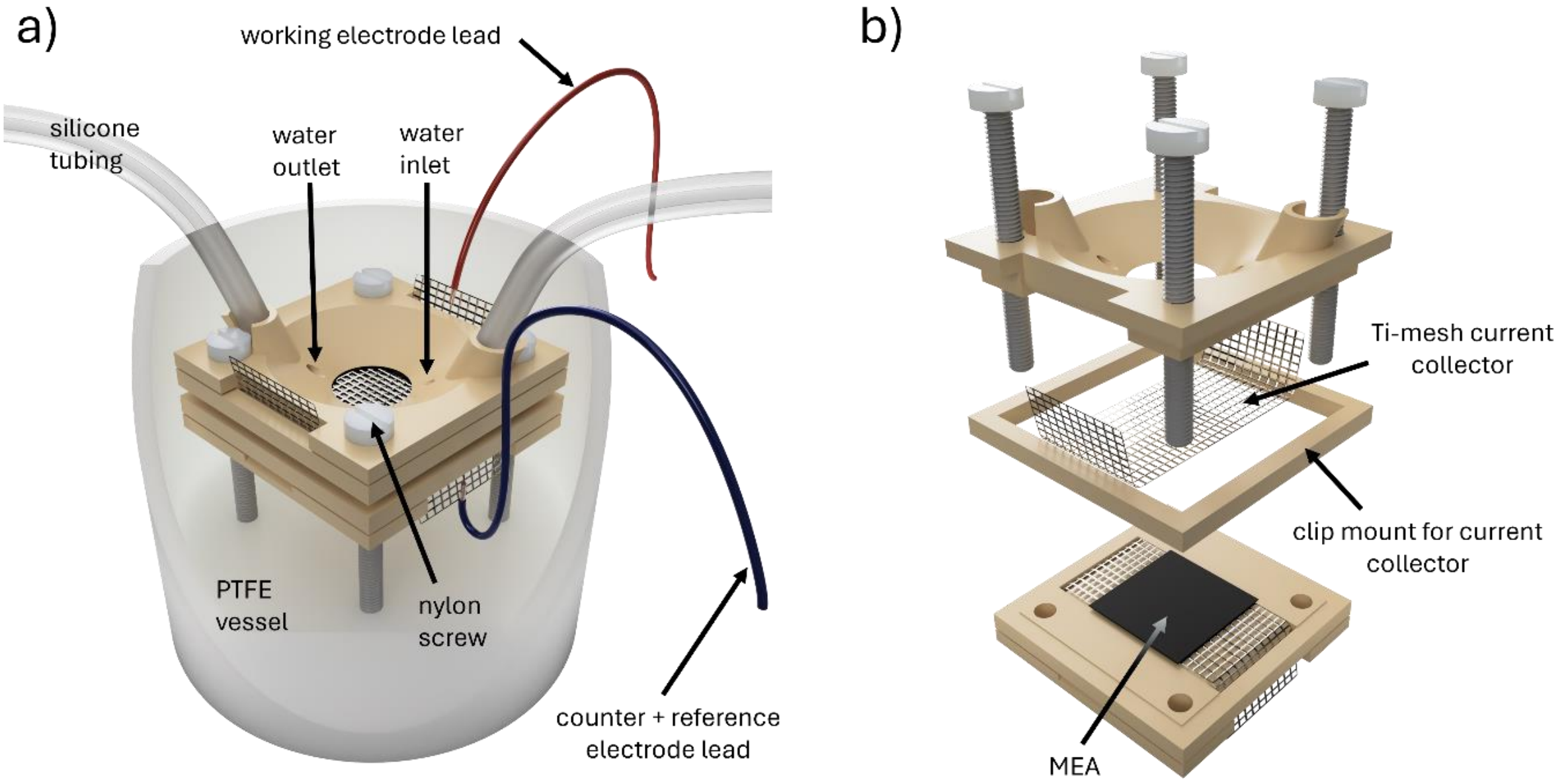

Fig. S2: Rendered 3D model of the utilized electrolysis setup. Fully assembled setup (section in PTFE vessel removed for clarity) (a); Exploded view of the MEA mount (b).

Technical drawing of cell setup

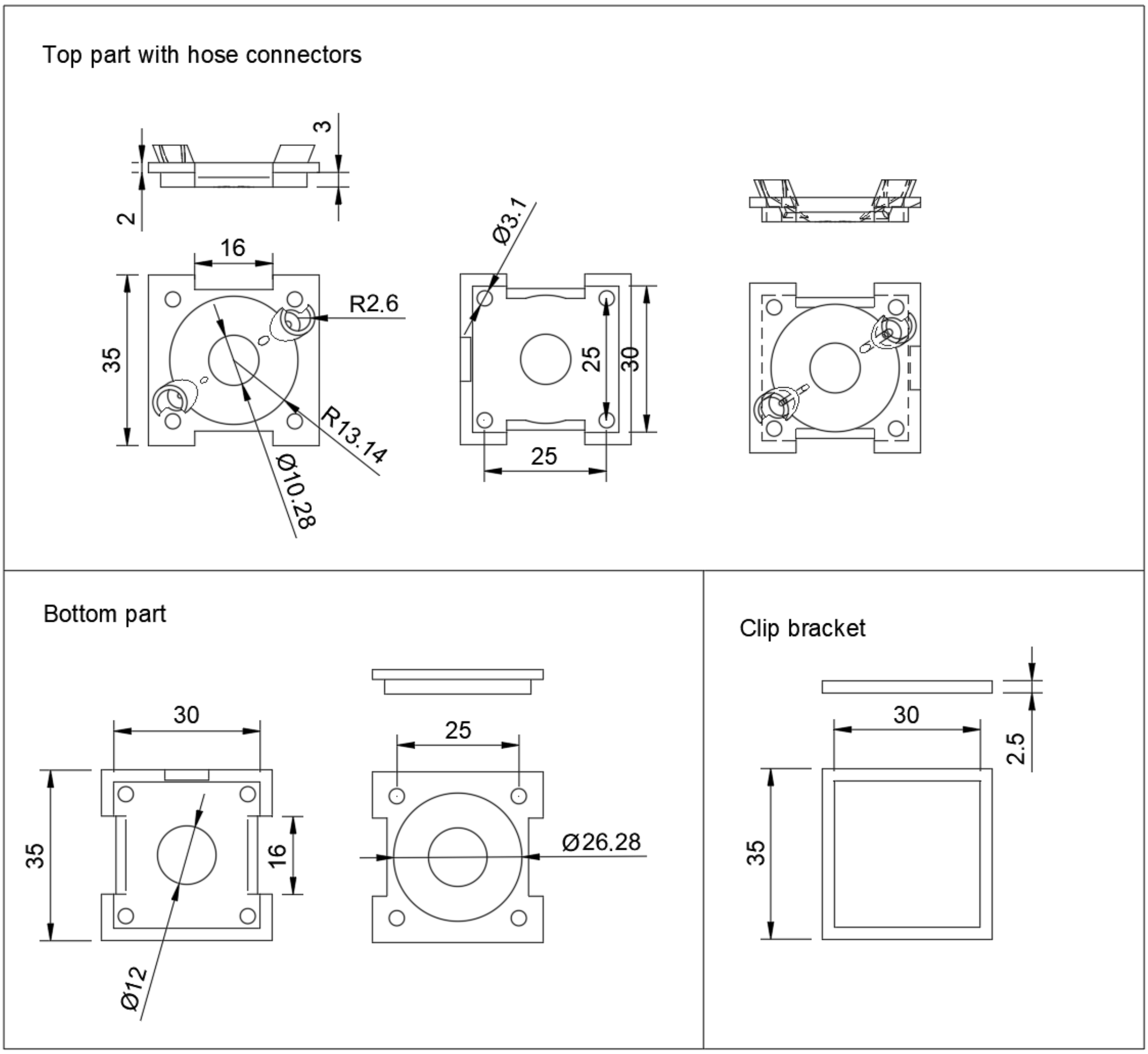

Fig. S3: Technical drawing of the top and bottom parts, as well as the bracket used for mounting the current collector mesh.

Electrochemical sequence

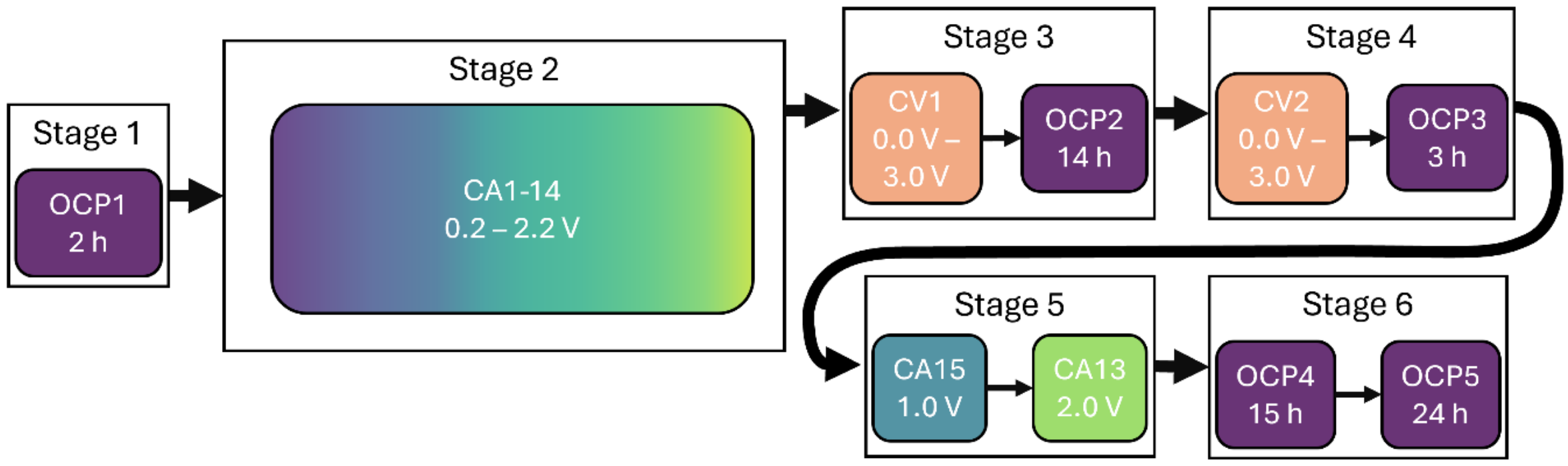

Fig. S4: Sequence of electrochemical experiments.

Potential sequence of CA1-14

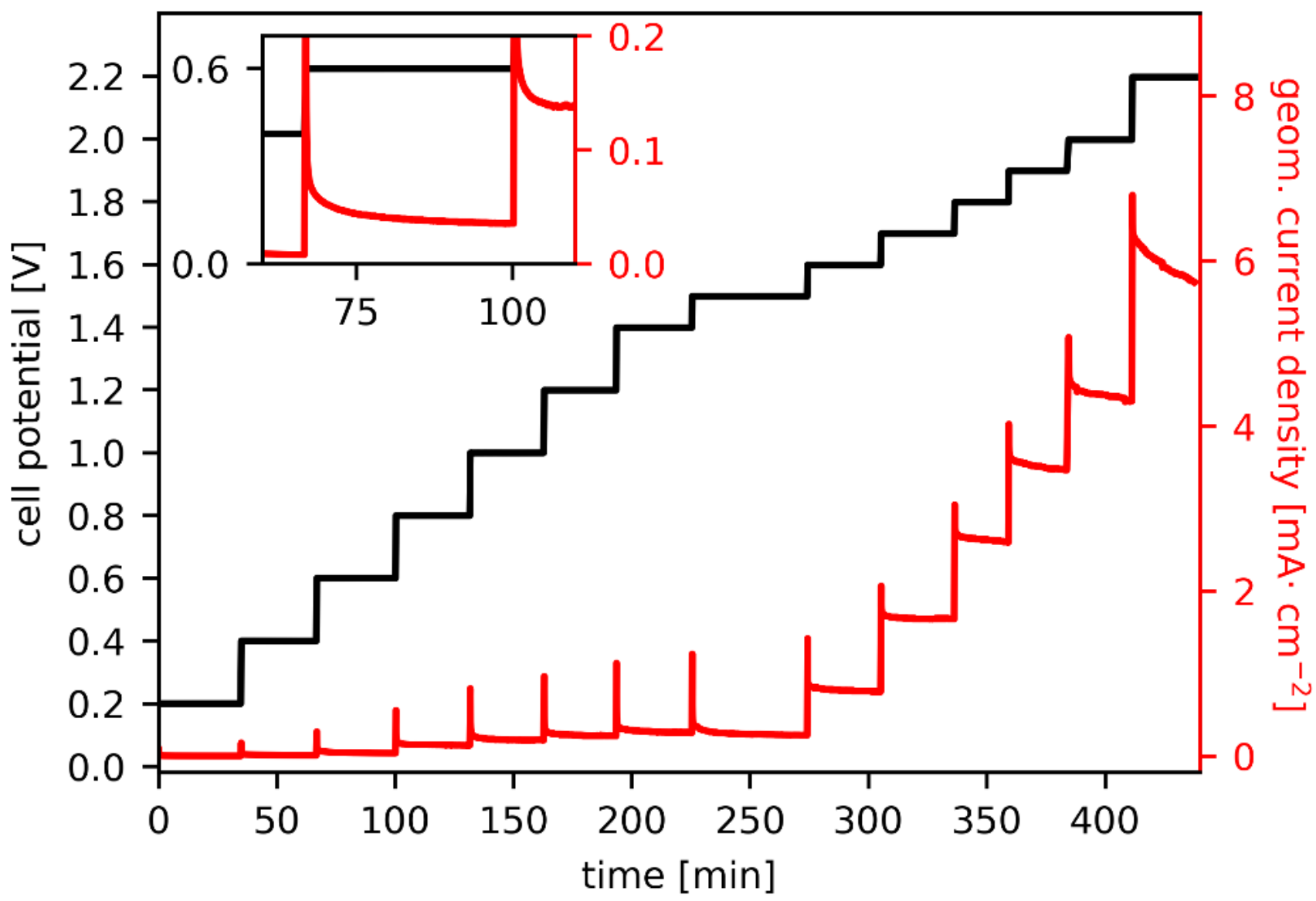

Fig. S5: Voltage profile and current response during the *in operando* experiment.

It is evident that the geometrical current density spikes at the start of each potential step, rapidly decreasing over the next 5 minutes and reaching close to constant values after 10 minutes. Starting from 1.9 V the time dependent current response starts to be significantly less rapid and at 2.2 V the geometrical current density does not reach its equilibrium even after 20 minutes.

XRD analysis

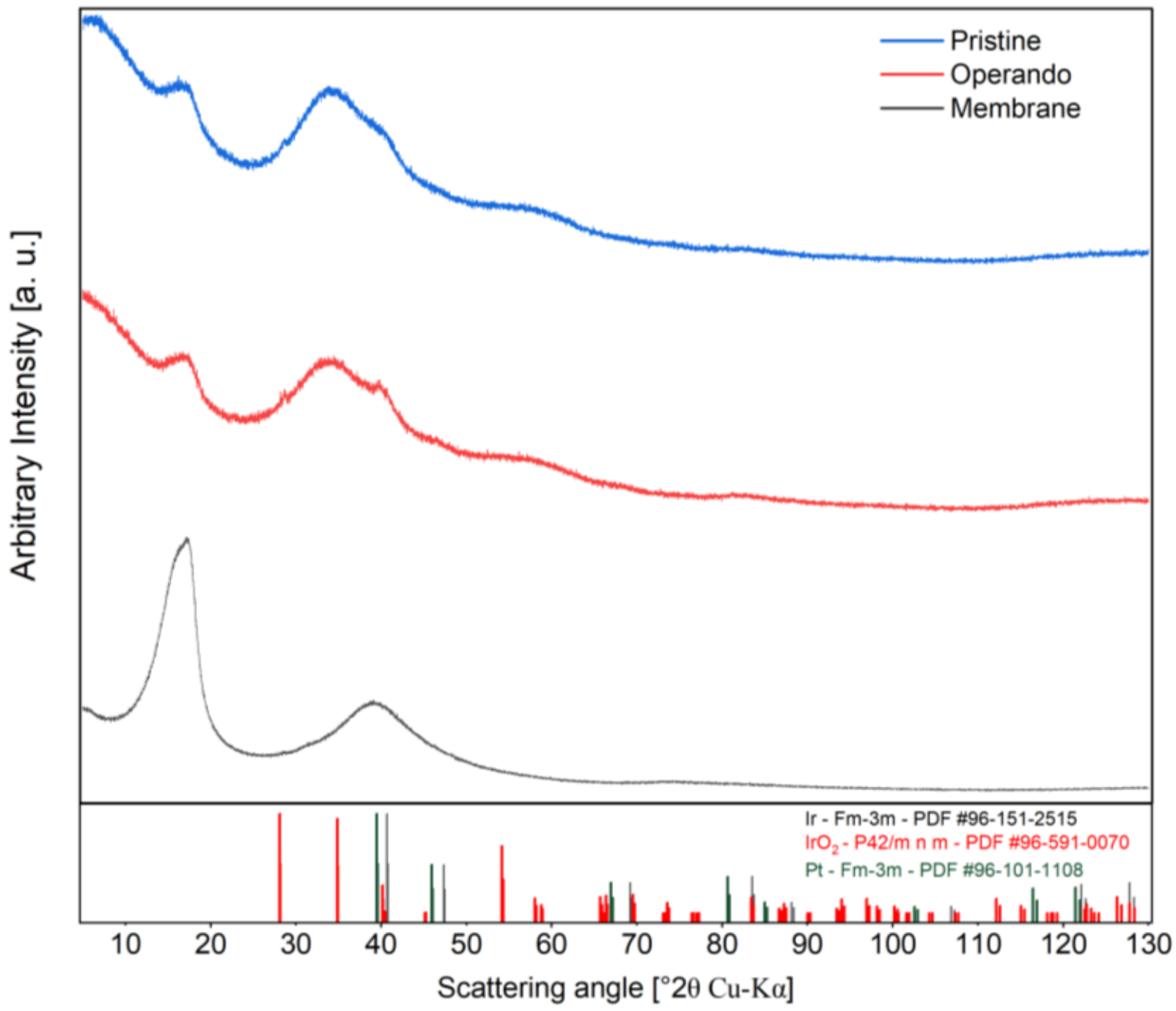

Fig. S6: XRD diffractograms of the pristine and operated anode, as well as a pure Nafion membrane for reference. Calculated reflexes of Ir, IrO_2 and Pt are displayed at the bottom.

X-ray diffraction (XRD) measurements were performed in reflection mode using a Malvern Panalytical (UK) Empyrean diffractometer equipped with Cu Kα radiation (λ = 1.5406 Å). Diffractograms were recorded over an angular range of 5–130° 2θ with a total acquisition time of 5 h per sample.

Both the pristine and operated sample show broad reflexes that can be assigned to iridium oxide based on their position. The broad nature of the reflexes suggest that the iridium oxide is in an amorphous form. The additional sharper reflex visible in the diffractogram of the operated sample at ~39° is assigned to Pt, which was utilized to coat the current collector mesh and left visible bright metallic marks on the anode after operation. Otherwise no significant differences can be observed between both samples, suggesting that the structural changes observed in the *in operando* Raman experiment are largely reversible.

Cyclic voltammetry

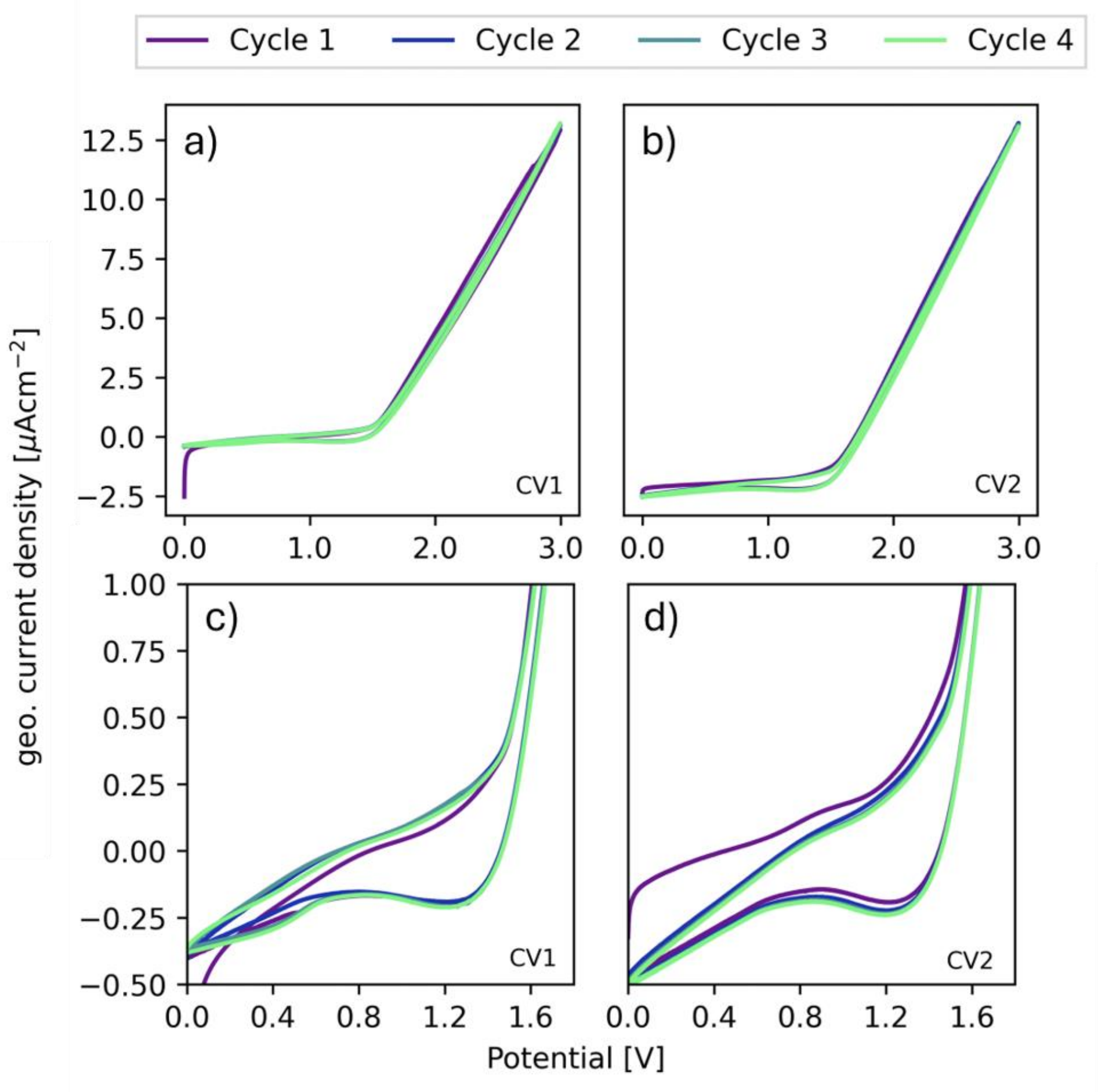

Fig. S7: Cyclic voltammetry experiments during stage 3 and 4. CVs 1 (a,c) and 2 (b,d). Whole potential range (a,b); zoomed-in plots (c,d). Four cycles were performed between 0.0 V and 3.0 V with a scan rate of 20 mVs^{-1}. The potential was cycled back to 0.0 V in the last cycle.

OCP4

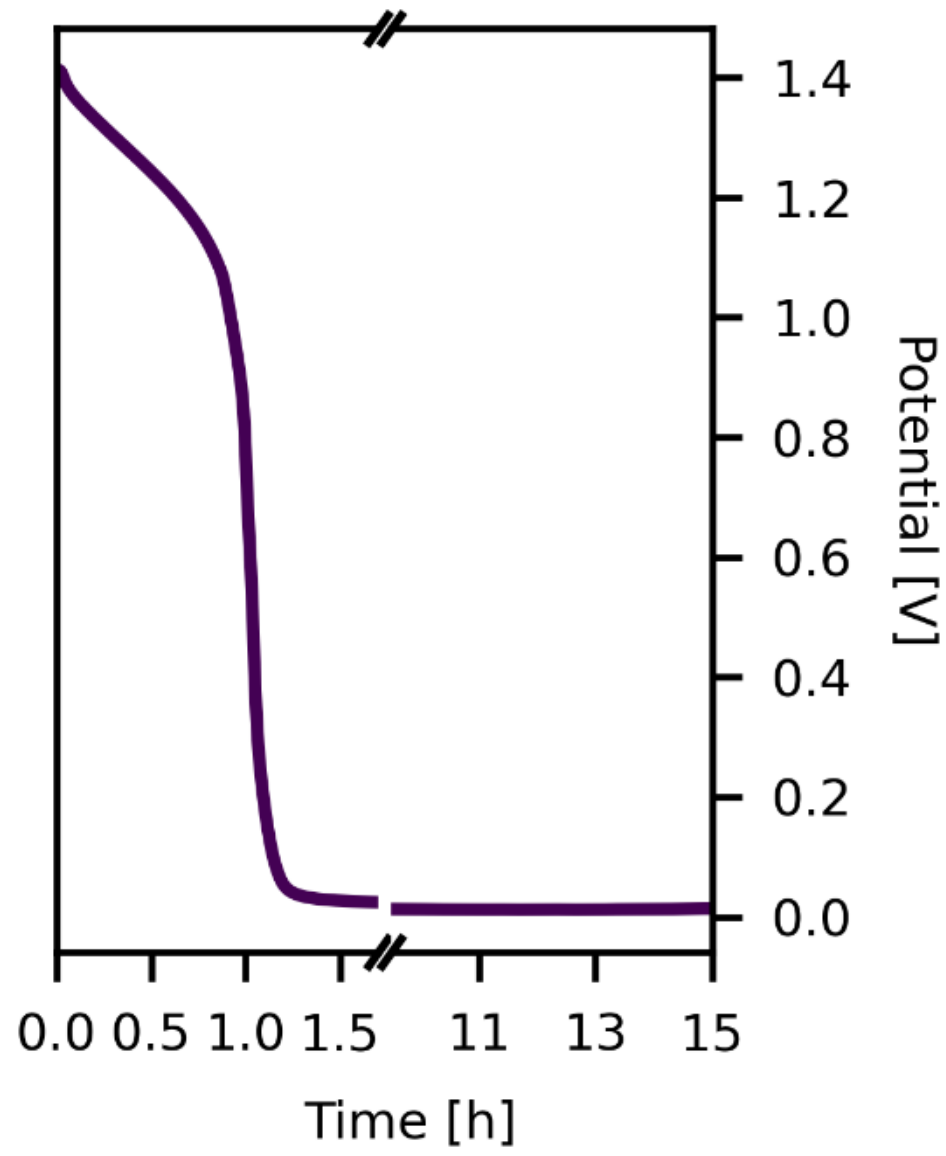

Fig. S8: Cell potential at open circuit. The cell potential drop in the OCP4 in the first 1.5 h likely predominantly stems from the cathode side as observed by Brightman et al.[3]

While the drop in cell potential that is observable for OCP4 in the first 1.5 h (Fig. S7) might implicate a significant alteration of the OER catalyst, contributions from the cathode side dominate the cells OCP as observed by Brightman et al.[3] Therefore, the observed changes in the Raman signature are most likely unrelated to the observed potential drop

Single non-averaged spectra

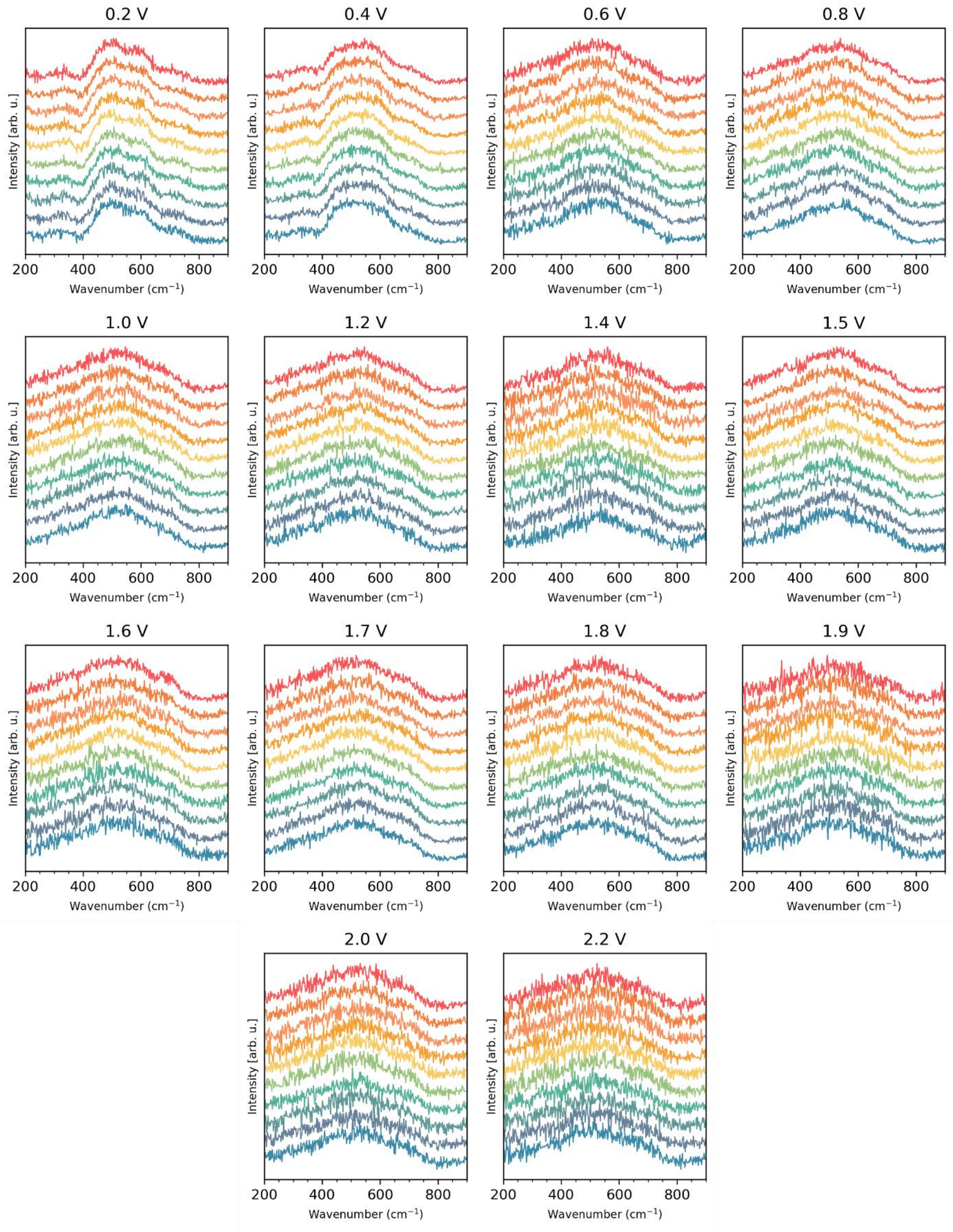

Fig. S9: Individual spectra acquired at each potential step. The absence of spectral evolution within each set indicates that measurements were performed after the system had reached a quasi-steady state following the applied potential change.

Catalyst states and reset

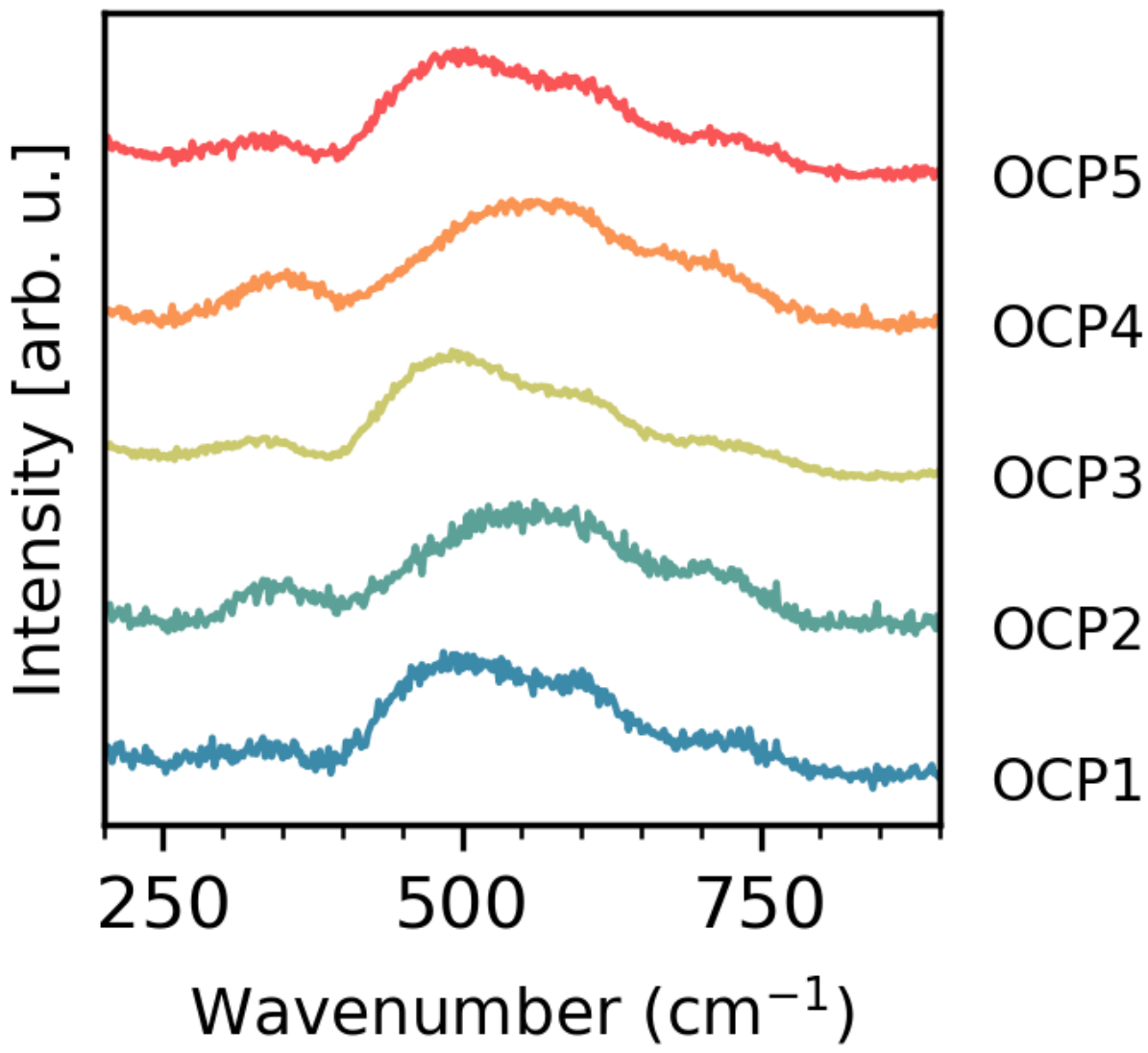

Fig. S10: Raman spectra acquired at OCP steps during the experimental procedure.

The Raman spectrum recorded at OCP2, approximately 14 hours after the first potential cycling sequence, exhibits a markedly higher Ir^{3+}/Ir^{4+} ratio compared to the pristine catalyst. This is visible by the shift of the maximum intensity to higher wavenumbers due to a higher contribution of the δ band at ~600 cm^{-1}. The subsequent potential cycling step prior to OCP3 was performed to reset the catalyst to a state comparable to the initial condition, in line with literature reports that cycling replenishes IrO_x activity via rehydration of condensed oxide phases.[4–6] The Raman spectrum recorded at OCP3 closely resembles that at OCP1, confirming that the catalyst had largely returned to its initial state and demonstrating the reversibility of the observed spectral changes.

Development of the fitting model

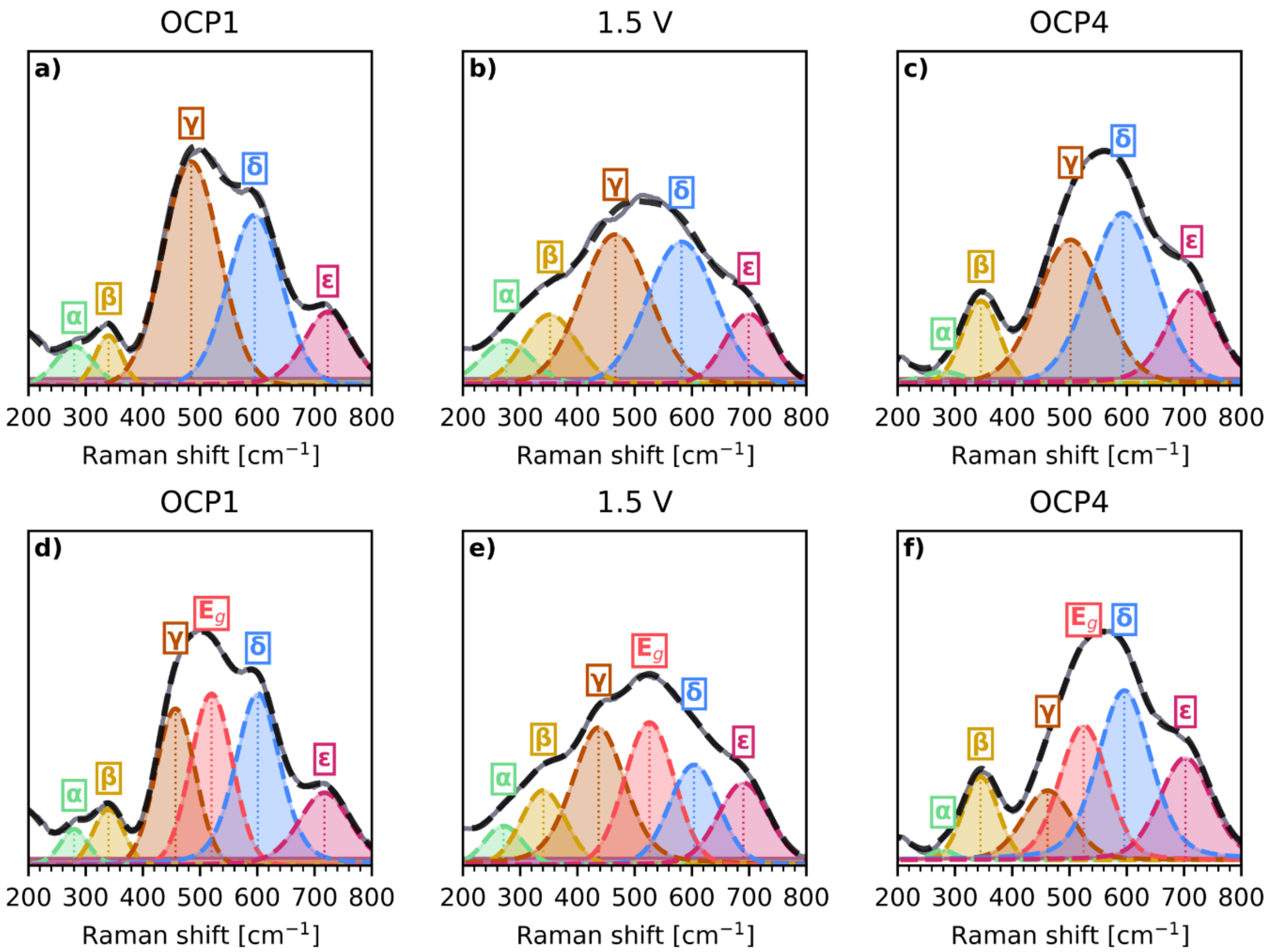

Fig. S11: Fits of Raman spectra at OCP and 1.5 V using models IrO_x_5P (a,b,c) and IrO_x/IrO_2_6P (d,e,f). The grey line depicts the experimental data, the black line is the sum of the fitted peaks which are displayed in the respective color of the assigned symbol.

The fitting model for the evolution of iridium oxo hydroxides based on the study of Pavlovic et al.[1] utilizes the five components α, β, γ, δ, and ε. It is referred to as "IrOx_5P" from now on indicating the type of oxide and the 5 peaks α-ε considered in the model. Representative fits are depicted in Fig. S8a-c. Here, γ and ε redshift with increasing potential, while the other peak positions are fixed. An additional peak around 180 cm^{-1} that is observable in the experiments was also included in all models to improve the fitting procedure. The γ peak shifts to considerably higher wavenumbers after 15 h of OCP4 in stage 6. This contradicts the interpretation of Pavlovic et al. where the shift was explained by a phenomenon similar to Stark tuning. Stark tuning describes the shift of spectral lines due to an increased electric field created by charging of a crystal structure, similar to the Zeeman effect for nuclear spins in a magnetic field.[1] A blue shift of γ as observed in Fig. S8c, however, suggests a decrease in electric field strength at OCP4 compared to OCP1.

Pavlovic et al. describe the formation of IrO_2 with increasing potential, depending on the type of IrO_x particle that was observed.[1,7] Even though a E_g peak is not evident in the spectra, fitting

the peaks α-ε without inclusion of rutile IrO_2 components results in a high deviation of the fit, as presented in Fig. S8a-c. In particular at cell potentials larger than 0.4 V, the intricate shoulder of the feature at 440 cm^{-1} is not sufficiently represented by only five components. Thus, the fitting model was expanded by an additional E_g band of rutile IrO_2, now referred to as "IrO_x/IrO_2_6P".[1,2,8] Overall, the addition of a sixth peak results in sufficient description of the system for every applied cell potential as depicted in Fig. S8d-f. With an increase in cell potential, the redshift of the γ and ε bands is evident, as well as an intensity decrease of δ and an intensity increase of γ. Furthermore, instead of a blue shift of the γ signal for OCP4 (Fig. S8f), the intensity of the band now decreases, which results in the observed shift of the broad feature to higher wavenumbers. Model IrO_x/IrO_2_6P generally exhibits improved fitting of the IrO_x signal, thus the existence of an IrO_2 phase within the material is likely.

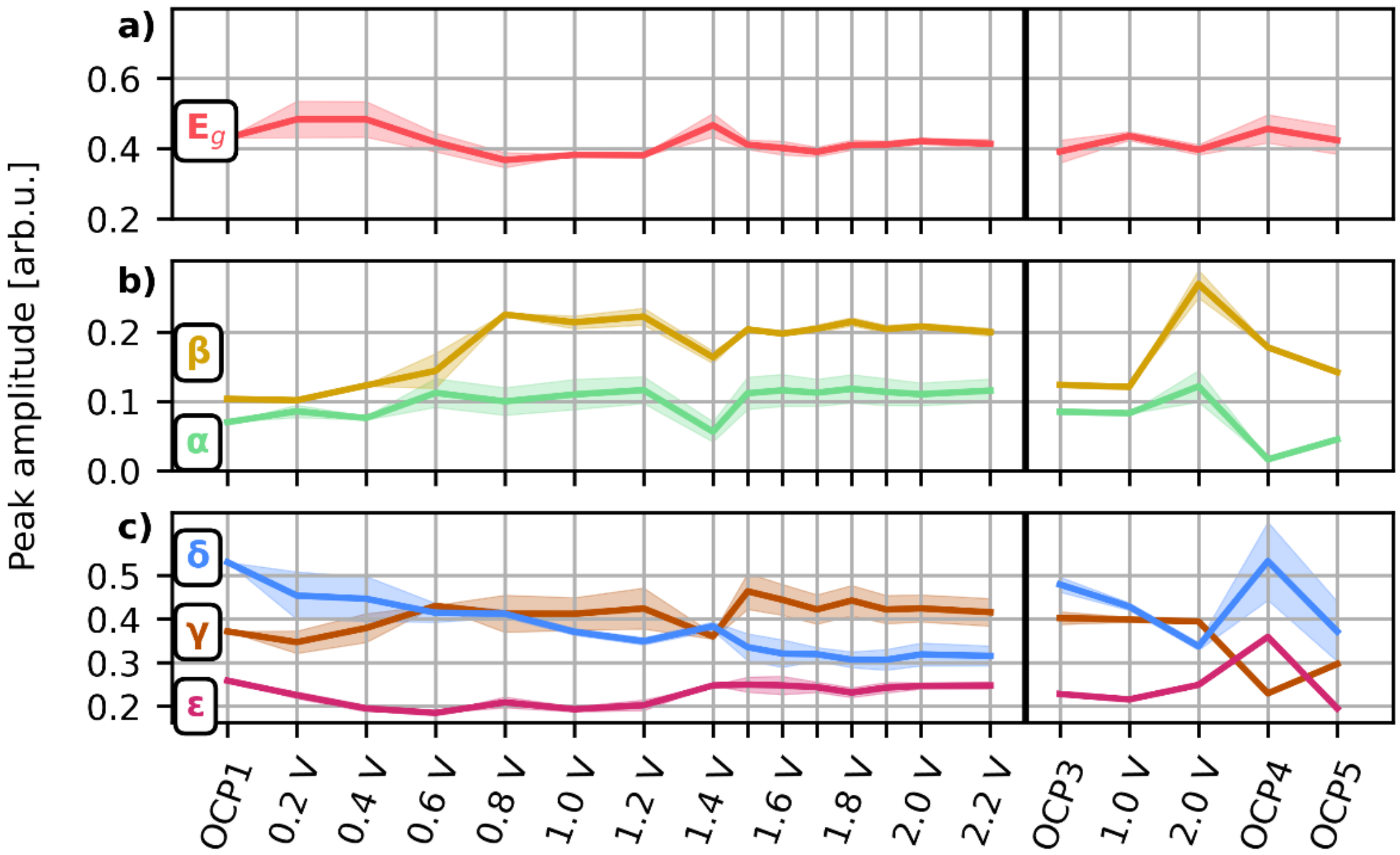

Fig. S12: Peak amplitudes of the individual components, derived from the deconvolution of the spectra for the respective applied potentials and OCPs using the assignments proposed by Pavlovic et al. E_g band of IrO_2 (a); Peaks assigned to Ir^{4+}-O-Ir^{4+} twisting vibrations (b); Peaks assigned to Ir^{4+}-O-Ir^{4+} (γ and ε) and Ir^{4+}-O-Ir^{3+} (δ) stretching vibrations (c). Values are averaged over 10 runs of the randomized fitting procedure. Standard deviations are represented as the shaded areas below and above each line.

Fig. S9 depicts the respective peak amplitudes resulting from the fitting procedure. It is evident that the most significant alteration of the spectra happens at cell potentials below 0.8 V. The E_g band initially increases but starts to decrease at 0.6 V. The peaks associated with Ir^{4+}-O-Ir^{4+} twisting vibrations α and β increase in the same range as well as the peak γ, which is associated with the Ir^{4+}-O-Ir^{4+} stretching vibration. Contrarily, the δ band associated with the Ir^{4+}-O-Ir^{3+} stretching vibration diminishes at the same cell potentials. These trends in the change of amplitudes of peaks α-δ are most pronounced moving from 0.4 V to 0.6 V. Peak ε however,

which is also associated with the Ir^{4+}-O-Ir^{4+} stretching vibration, diminishes in the same potential range.

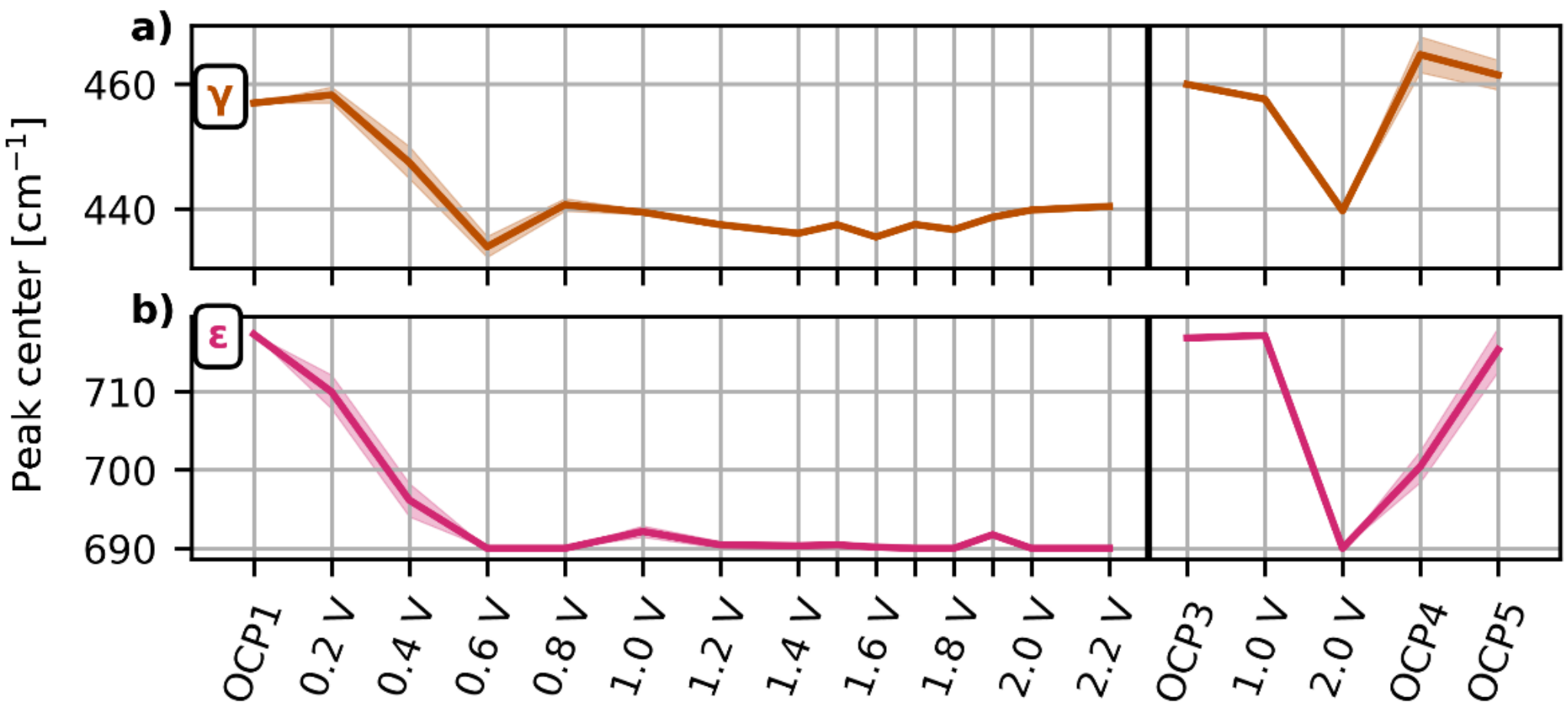

Fig. S13: Positions of peaks γ (a) and ε (b) during the in-operando experiment. Values are averaged over 10 runs of the fitting procedure.

Peaks γ and ε show a significant red shift in this range, indicating the charging of the structure, as suggested by Pavlovic et al. (Fig. S10). Thereby, both peaks can be identified as $Ir^{4.x+}$ species from 0.8 V on. After 0.8 V the signal δ associated with Ir^{3+} diminishes further, while most of the other signals stay constant within margin of errors. The E_g band and ε peak increase, regaining close to their initial amplitudes at 1.4 V.

At 1.4 V then, the aforementioned dip in intensity in the range of 200 cm^{-1} to 400 cm^{-1} is evident for peaks α, β, and γ, while peaks E_g, δ and ε increase in amplitude slightly. This behavior is also shown in stage 5, exhibiting similar trends in the evolution of the amplitudes upon increase of the cell potential to 2.0 V. After 15 h of OCP, peaks δ and ε show a significant increase in amplitude, surpassing their values before potential increase significantly, while peaks α and γ decrease to a similar extent reaching values significantly lower than at OCP1. β decreases, still retaining a higher amplitude than before the potential application.

In general, it can be stated that Ir^{3+} oxidation states are oxidized to Ir^{4+} and higher with increasing cell potential. This is indicated by the diminishing δ peak, concerted increase of peaks associated with Ir^{4+}, as well as the red shift of peaks γ and ε indicating charging of the structure. The E_g band of rutile IrO_2 shows a more variable behavior, initially indicating formation of IrO_2 during potential increase to 0.4 V which can be explained by the sudden increase in Ir^{4+} concentration.

Amorphization follows moving towards 1.0 V. Subsequently IrO_2 forms as suggested by Pavlovic et al., reaching maximum concentration at 1.4 V. It then settles at values similar to those of the pristine catalyst. In the Raman spectrum for OCP4, concerted increase of the amplitudes of δ and ε but decrease in amplitude of γ is evident. This observation is contradictory to the statement that γ and ε are associated with the same Ir^{4+} center.

To address the aforementioned discrepancies, model IrO_x/IrO_2_6P was expanded based on the peak assignments of Saeed et al.[2] The ζ band is not observable in the experiments and is therefore not assessed. Additionally implementing the θ band in the fitting model results in mostly the same potential dependent response of the peak amplitudes with the exception of the ε peak. Contrary to IrO_x/IrO_2_6P, its amplitude remains at a low level after its decrease from OCP1 to 0.6 V, while simultaneously the signal θ increases in this range. For this model "IrO_x/IrO_2_7P", this change in response reflects the presence of higher iridium oxidation states that affect the Ir-O-Ir stretching vibrations.

To further expand on the assignments of Saeed et al. represented in model IrO_x/IrO_2_7P, which handles the redshifted ε peak as a separate component, a new ι peak was implemented instead of red shifting the γ peak. Various models including the α, β, δ, γ, ε, θ and ι peaks have been tested, mostly altering the implementation of the IrO_2 E_g band. Less restrictive boundaries on the E_g shift resulted in Raman shifts that were significantly lower than the commonly reported values, but more sensible changes in amplitudes during potential variation. Therefore, this model (IrOx/IrO2_8P) was used as the final fitting model for further discussion and all spectra are shown in Fig S14.

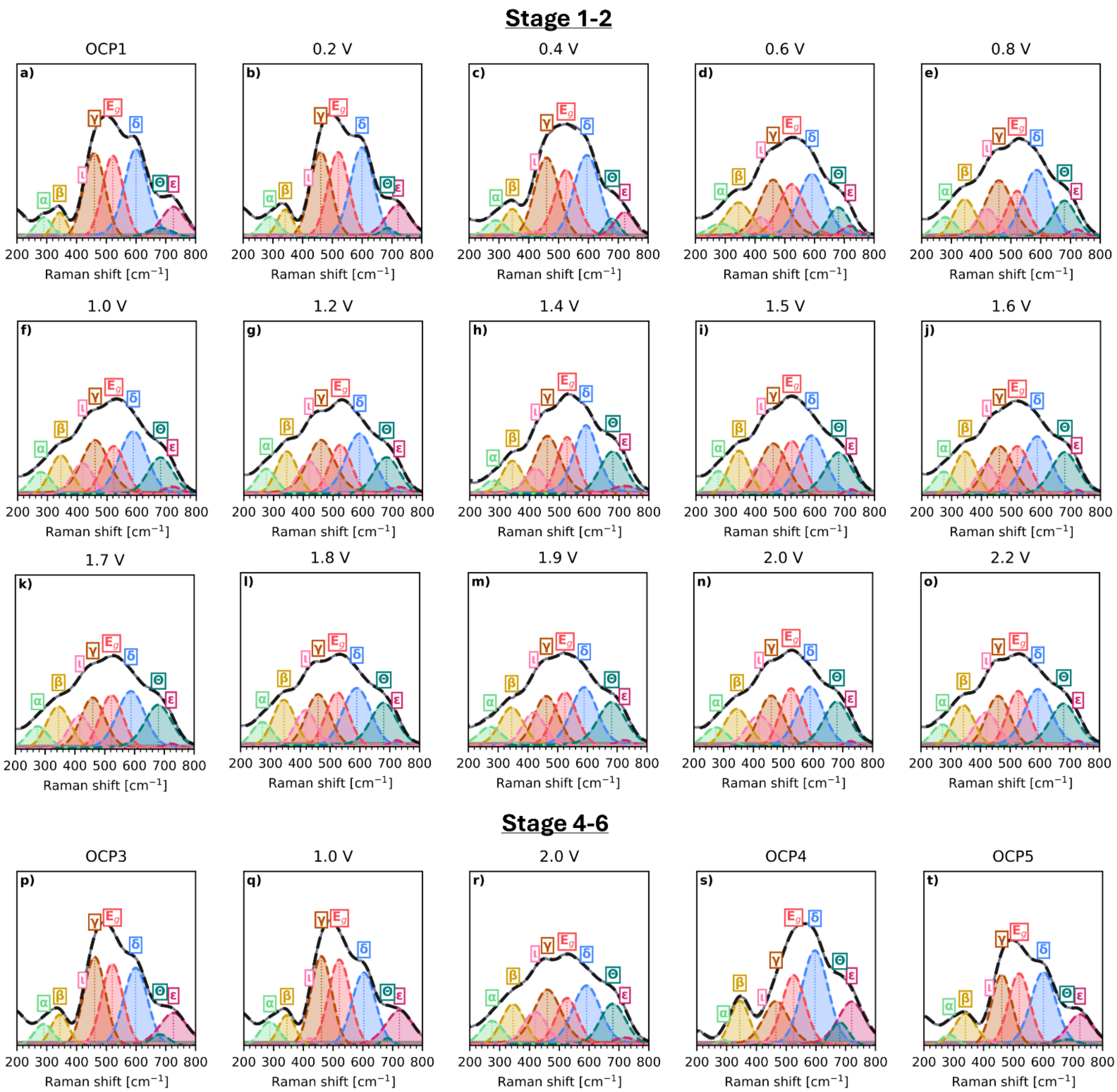

Fig. S14: Fits of the *in operando* Raman spectra for stages 1 and 2 (a-o) and stages 4-6 (p-t) utilizing fitting model IrO_x/IrO_2_8P

Two-dimensional correlation analysis

The dynamic spectrum $\tilde{y}(\tilde{\nu}, E)$ is computed as the deviation from a reference spectrum $\bar{y}(\tilde{\nu})$ as seen in equation S1:

$$\tilde{y}(\tilde{\nu}, E) = \begin{cases} y(\tilde{\nu}, E) - \bar{y}(\tilde{\nu}) for\ E_{min} \leq E \leq E_{max} \\ 0 \quad otherwise \end{cases} \tag{S1}$$

$$\bar{y}(\tilde{\nu}) = y(\tilde{\nu}, OCV)$$

E: Cell potential

$\tilde{y}$: Dynamic spectrum

y: Spectrum

$\bar{y}$: Reference spectrum

E_{min}: Lowest potential

E_{max}: Highest potential

The reference spectrum is often chosen as the average of the spectra over the course of the employed perturbation, but other forms of reference spectra are viable. In this case, the spectrum at OCP1 is chosen instead of the potential average. In the original method proposed by Noda the spectra undergo Fourier transformation to decompose the potential-domain-signal into its constituent frequencies, each corresponding to a rate of change in the intensity at a spectral variable $\tilde{\nu}_a$ (equation S2).

$$\begin{aligned} \tilde{Y}_a(\omega) &= \int_{-\infty}^{\infty} \tilde{y}(\tilde{\nu}_a, E) e^{-i\omega E} dE \\ &= \tilde{Y}_a^{Re}(\omega) + i\tilde{Y}_a^{Im}(\omega) \end{aligned} \tag{S2}$$

$\tilde{Y}_a$: Fourier transform of the intensity variations at $\tilde{\nu}_a$

$\tilde{Y}_a^{Re}$: Real part of the fourier transform

$\tilde{Y}_a^{Im}$: Imaginary part of the fourier transform

ω: Frequency of intensity variation

The Fourier transform of the intensity variations at spectral variable $\tilde{\nu}_b$ is given as follows:

$$\tilde{Y}_b^*(\omega) = \int_{-\infty}^{\infty} \tilde{y}(\tilde{\nu}_b, E) e^{+i\omega E} dE \qquad \text{S3}$$
$$= \tilde{Y}_b^{Re}(\omega) - i\tilde{Y}_b^{Im}(\omega)$$

The correlation intensity between two spectral positions $\tilde{\nu}_a$ and $\tilde{\nu}_b$ can be obtained by equation S4:

$$\Phi(\tilde{\nu}_a, \tilde{\nu}_b) + i\Psi(\tilde{\nu}_a, \tilde{\nu}_b) \qquad \text{S4}$$
$$= \frac{1}{\pi(E_{max} - E_{min})} \int_0^{\infty} \tilde{Y}_a(\omega) \cdot \tilde{Y}_b^*(\omega)\, d\omega$$

Φ: Real part of the correlation intensity

Ψ: Imaginary part of the correlation intensity

The real and imaginary parts $\Phi(\tilde{\nu}_a, \tilde{\nu}_b)$ and $\Psi(\tilde{\nu}_a, \tilde{\nu}_b)$ in equation S4 are the synchronous and asynchronous correlation spectra. They depict the similarity and dissimilarities of the potential dependant behaviour of spectral intensity measured at the Raman shifts $\tilde{\nu}_a$ and $\tilde{\nu}_b$.

Noda later proposed an improved method for computing these correlation spectra circumventing the use of a Fourier transformation, making it computationally more efficient and easier to apply.[9] A detailed mathematical derivation can be found in Nodas work. The synchronous correlation intensity matrix can be calculated as follows:

$$\Phi(\tilde{\nu}_a, \tilde{\nu}_b) = \frac{1}{m-1} \tilde{y}(\tilde{\nu}_a,)^T \tilde{y}(\tilde{\nu}_b) \qquad \text{S5}$$

Essentially the synchronous correlation intensity is the potential average of the dynamic intensity variations measured at $\tilde{\nu}_a$ and $\tilde{\nu}_b$. A more sophisticated approach is needed for the computation of the asynchronous spectrum. Based on the Hilbert transformation Noda proposed the following formula to compute the intensity matrix for the asynchronous spectrum:

$$\Psi(\tilde{\nu}_a, \tilde{\nu}_b) = \frac{1}{m-1} \tilde{y}(\tilde{\nu}_a,)^T N \tilde{y}(\tilde{\nu}_b) \qquad \text{S6}$$

N: Hilbert-Noda transformation matrix

In which N is the Hilbert-Noda transformation matrix which is described in equation S7:

$$N = \begin{cases} 0 & if \quad j = K \\ 1/\pi(k-j) & otherwise \end{cases} \qquad \text{S7}$$

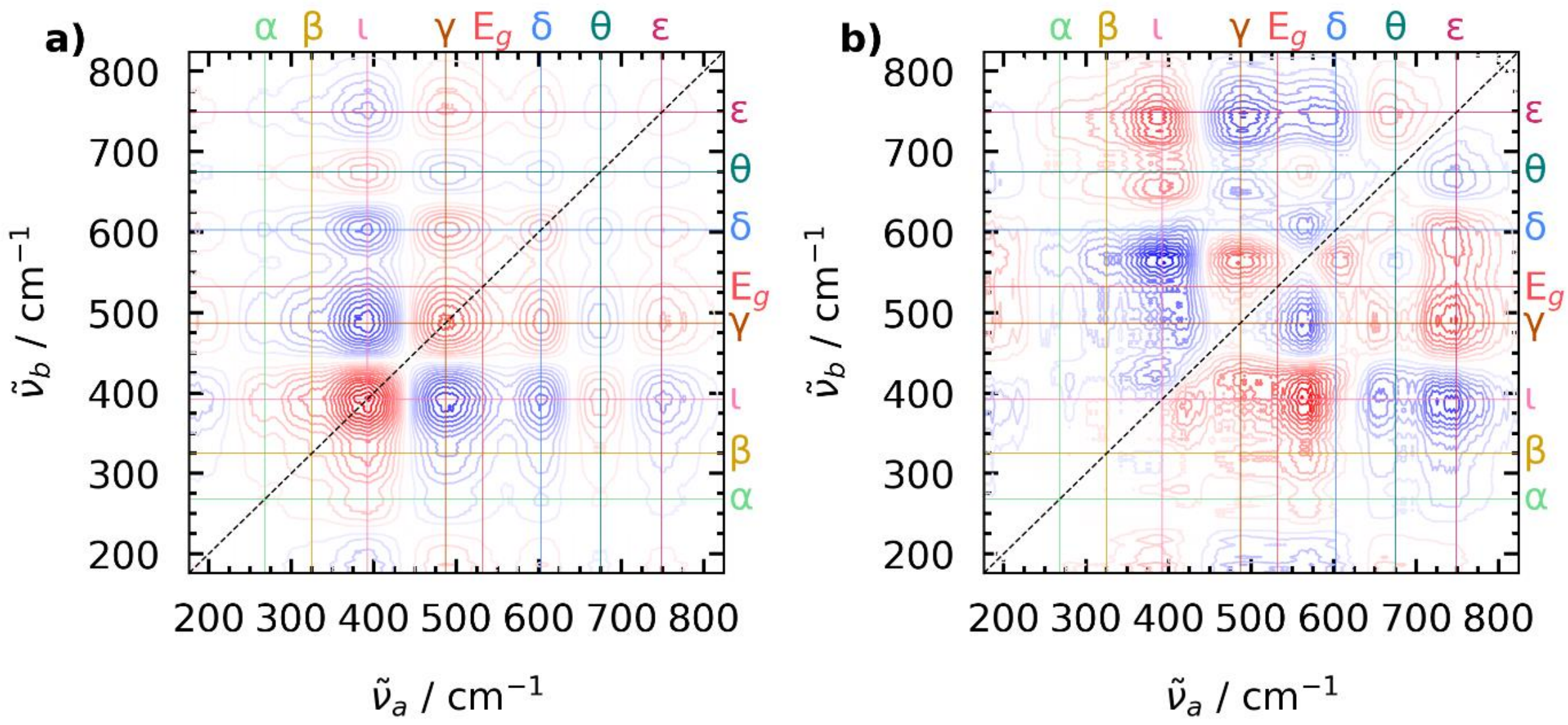

Fig. S15: Synchronous (a) and asynchronous (b) correlation spectra of stages 1-2 of the *in operando* series. Positive values are depicted in red, negative values are depicted in blue. Peak positions as determined from the synchronous correlation spectrum are depicted using colored vertical and horizontal lines.

The synchronous spectrum is discussed in the main text of the publication. The asynchronous spectrum is less conclusive than the synchronous spectrum. It depicts asynchronous changes on the potential axis, i.e. meaning one peak increasing at lower potential, followed by the other at higher potential. Peak positions roughly correlate with the ones in the synchronous spectrum only if positioned further away from the diagonal, which might be caused by increased inaccuracy of band positions close to the diagonal as previously reported by Gericke et al. In general, even slight variations in peak shifts or widths can cause additional cross peaks.[10] Also, most of the intensity changes between the peaks correlate mostly synchronously as seen from the amplitudes received from the fitting procedure in Fig. 3d, suggesting that many peaks in the

asynchronous spectrum might originate from other effects. It is imaginable, that anomalous peaks could arise easily in asynchronous spectra of such complex systems consisting of many overlapping and dynamically changing peaks. Nevertheless, cross peaks of the ε peak all show matching positions with the peaks in the synchronous spectrum.

1. Pavlovic, Z., Rajan, C., Gao, Q., van Gastel, M. & Schlogl, R. Probing the Structure of a Water-Oxidizing Anodic Iridium Oxide Catalyst using Raman Spectroscopy. *ACS Catal.* **6**, 8098–8105 (2016).
2. Saeed, K. H., Forster, M., Li, J. F., Hardwick, L. J. & Cowan, A. J. Water oxidation intermediates on iridium oxide electrodes probed by in situ electrochemical SHINERS. *Chemical Communications* **56**, 1129–1132 (2020).
3. Brightman, E., Dodwell, J., van Dijk, N. & Hinds, G. In situ characterisation of PEM water electrolysers using a novel reference electrode. *Electrochem. commun.* **52**, 1–4 (2015).
4. Papakonstantinou, G., Spanos, I., Dam, A. P., Schlögl, R. & Sundmacher, K. Electrochemical evaluation of the de-/re-activation of oxygen evolving Ir oxide. *Physical Chemistry Chemical Physics* **24**, 14579–14591 (2022).
5. Pickup, P. G. & Birss, V. I. A model for anodic hydrous oxide growth at iridium. *J. Electroanal. Chem. Interfacial Electrochem.* **220**, 83–100 (1987).
6. Zlatar, M. *et al.* On the growth and water oxidation stability of hydrous iridium oxide. *EES Catalysis* **3**, 521–534 (2025).
7. Pavlovic, Z., Ranjan, C., van Gastel, M. & Schlogl, R. The active site for the water oxidising anodic iridium oxide probed through in situ Raman spectroscopy. *Chemical Communications* **53**, 12414–12417 (2017).
8. Huang, Y. S. *et al.* Raman spectrum of IrO2. *Solid State Commun.* **70**, 517–522 (1989).
9. Noda, I. Determination of Two-Dimensional Correlation Spectra Using the Hilbert Transform. *Appl. Spectrosc.* **54**, 994–999 (2000).
10. Gericke, A., Gadaleta, S. J., Brauner, J. W. & Mendelsohn, R. Characterization of biological samples by two-dimensional infrared spectroscopy: Simulation of frequency, bandwidth, and intensity changes. *Biospectroscopy* **2**, 341–351 (1998).
